# Emergence of co-tuning in inhibitory neurons as a network phenomenon mediated by randomness, correlations, and homeostatic plasticity

**DOI:** 10.1126/sciadv.adi4350

**Published:** 2024-03-20

**Authors:** Fereshteh Lagzi, Adrienne L. Fairhall

**Affiliations:** ^1^ Department of Physiology and Biophysics, University of Washington, 1705 NE Pacific Street, Seattle, WA 98195-7290, USA.; ^2^ Computational Neuroscience Center, University of Washington, 1705 NE Pacific Street, Seattle, WA 98195-7290, USA.

## Abstract

Cortical excitatory neurons show clear tuning to stimulus features, but the tuning properties of inhibitory interneurons are ambiguous. While inhibitory neurons have been considered to be largely untuned, some studies show that some parvalbumin-expressing (PV) neurons do show feature selectivity and participate in co-tuned subnetworks with pyramidal neurons. In this study, we first use mean-field theory to demonstrate that a combination of homeostatic plasticity governing the synaptic dynamics of the connections from PV to excitatory neurons, heterogeneity in the excitatory postsynaptic potentials that impinge on PV neurons, and shared correlated input from layer 4 results in the functional and structural self-organization of PV subnetworks. Second, we show that structural and functional feature tuning of PV neurons emerges more clearly at the network level, i.e., that population-level measures identify functional and structural co-tuning of PV neurons that are not evident in pairwise individual-level measures. Finally, we show that such co-tuning can enhance network stability at the cost of reduced feature selectivity.

## Teaser

Inhibitory tuning can emerge as a network phenomenon resulting from randomness, correlations, and homeostasis.

## INTRODUCTION

It is unclear how neurons in the cortex develop feature tuning. In mice, excitatory neurons have relatively strong feature tuning; however, there has been a long debate about the tuning of inhibitory interneurons. Among different inhibitory subtypes in the cortex, which may play different and complementary roles in sensory processing (*1*, *2*), parvalbumin-expressing subtypes (PV) are thought to help shape the tuning responses of excitatory neurons to different features of the sensory input (*3*–*6*). Currently, there are two groups of seemingly conflicting studies about the tuning of PV neurons to pyramidal excitatory (E) neurons, i.e., covariation of E and PV firing rates (functional tuning) and organized patterns of connectivity between PV and E neurons (structural tuning) in mouse V1.

One group of studies has reported that despite the tuning of the E neurons to certain input features, such as orientation, individual PV neurons are not tuned or only weakly tuned to those input features (*7*–*11*), and thus concludes that PV neurons do not participate in co-tuned subnetworks with E neurons. In these studies, a weak average pairwise correlation between E and PV neurons in response to a given input feature, such as a particular orientation, has been reported. Indeed, given the dense connectivity profile of E to PV neurons in layer 2/3 (*8*, *12*), as well as the salt and pepper organization of feature maps in mice (*13*), PV neurons might be expected to receive inputs from excitatory neurons tuned to many different features, therefore remaining broadly tuned and without feature-specific connections to excitatory neurons.

A second set of studies, however, shows that despite the salt and pepper organization of feature maps in mice, PV neurons can have a sharp tuning response (functional tuning) (*14*). Further, they can participate in subnetworks (motifs) of strongly coupled PV and E neurons with relatively strong reciprocal connections (structural tuning) (*14*–*17*). Therefore, in mice, PV tuning has been framed as related to the strength of the connections between E and PV neurons, which correlates with the response similarity between PV and E neurons (*16*). These reciprocal connections were found to show a relatively constant excitatory postsynaptic potential (EPSP) to inhibitory postsynaptic potential (IPSP) ratio; this relation depends on the activity of the E and PV neurons (*16*), and is mediated by layer 4 excitation (*15*, *18*). In these studies, conclusions about the existence of tuning were reached by considering network-level characterizations: in one case, the pattern of connectivity of neighboring excitatory neurons to individual PV neurons (*15*, *19*) and, in the other, the collective functional responses of PV neurons (*17*).

There is a conceptual difference between the “tuning” properties of individual neurons to a given input feature and the “co-tuning” of neurons. Tuning refers to the selectivity of individual neuron responses to specific input features; however, co-tuning considers the similarity between responses of two (or more) neurons given a feature in the input. Currently, it is not clear how weakly tuned PV neurons can form strongly co-tuned subnetworks with feature-tuned excitatory subnetworks. Here, we shed light on this possibility by first showing in a model that the tuning of inhibitory neurons could be an emergent network property rather than a built-in single-neuron phenomenon. Second, we highlight that because PV tuning is a network property, the emergence of tuned PV subnetworks can be better explained by population-level measures, while co-tuning may not be evident from pairwise measures.

To achieve this, we consider two facts and one hypothesis about layer 2/3 neurons in mouse V1. First, we take shared input from layer 4 to drive feature tuning and stimulus selectivity in excitatory subnetworks (*20*, *21*). Second, there is known to be considerable heterogeneity in the EPSPs that impinge on PV neurons (*8*, *12*, *22*). In addition, we assume that the homeostatic regulation of postsynaptic firing rate previously reported for GABAergic neurons (*23*–*26*) governs the synaptic dynamics of the connections from PV to E neurons. We will show that these considerations result in the emergence of co-tuned PV and pyramidal “subnetworks,” although only a small fraction of individual PV neurons are strongly tuned to pyramidal neurons, thus bridging the two groups of experimental reports on PV tuning.

Finally, we examine the dynamical consequences of this predicted network structure. We show theoretically that co-tuned PV to E subnetworks provide stability to the network dynamics and expand the dynamic range of frequency responses of the E neurons, but at the cost of reducing competition among excitatory assemblies, leading to reduced selectivity and reduced input amplification of the excitatory neurons.

## RESULTS

### Random connectivity from E to PV causes tuned PV to E connections

**Fig. 1.**
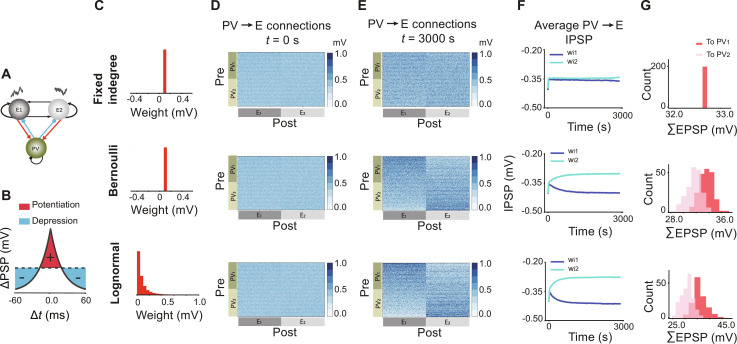
Effect of E to PV heterogeneity of connections on the emergence of tuned PV to E synaptic weights in a network with a single initial PV population. (**A**) Network schematic with two excitatory populations receiving independent shared correlated inputs. (**B**) Symmetric STDP rule for the inhibitory synapses onto excitatory neurons. (**C**) Three distributions from E to PV neurons: fixed indegree (FID), Bernoulli with a fixed EPSP amplitude, and an log-normal (L-N) distribution. (**D**) Before initiating the plasticity rule, the connectivity matrix for the synaptic weights (IPSP) from PV to E neurons was originally random with a fixed IPSP amplitude. PV neurons are sorted and labeled based on the labels in (E). (**E**) PV to E connectivity matrix at the end of the simulation, and PV index sorting for the networks with FID, Bernoulli, and L-N synaptic weight distributions, respectively. PV_1_ and PV_2_ subnetworks are defined based on the block structure of the emerging connectivity matrix. (**F**) Average IPSP amplitude for all connections from PV_1_ and PV_2_ neurons to E_1_ neurons. (**G**) Distribution of total EPSP projections from individual E_1_ neurons to PV_1_ and PV_2_ neurons, after assigning memberships to PV neurons.

To study how PV neurons develop their connections to E neurons, we considered simple network scenarios with random connections. As a starting point, we simulated networks with two excitatory assemblies (E_1_ and E_2_ in Fig. 1A) and one population of PV neurons. E neurons in different assemblies were taken to be coupled with synaptic strength *J*, representing the EPSP amplitude. However, supported by experimental evidence from (*21*, *27*), neurons within each excitatory assembly were interconnected with a stronger EPSP amplitude *wJ*, where *w* > 1. Excitatory neurons in each population received inputs from two sources: a shared correlated source with a firing rate of *c*η, which was private to each assembly and projected the same spike pattern to all neurons in each assembly—representing feature tuning in E neurons—and a common background source giving random and independent Poisson input with a firing rate of (1 − *c*)η. PV neurons were not tuned to any input feature or, equivalently, did not receive any shared correlated input. They were connected to one another with a constant IPSP amplitude and initially uniformly to all E neurons. However, connections from PV to E neurons were plastic, following the symmetric spike-timing dependent plasticity (STDP) rule proposed in (*23*, *25*), consistent with reports from layer 2/3 of visual cortex (*28*) and in OFC (*26*). According to this rule, a small timing separation between spikes of the presynaptic PV and the postsynaptic E neuron causes synaptic potentiation, while the firing of the PV neuron causes synaptic depression. This mechanism was demonstrated to drive firing rate homeostasis for the target E neurons [(*25*), Fig. 1B].

To study the effect of heterogeneity in the connections from E to PV neurons, we simulated networks with three different connectivity distributions with identical mean values. The simplest network had a single PV population, which received projections from each E assembly with a fixed in-degree (FID) distribution with identical EPSP amplitudes (Fig. 1C, top). The second network also had a fixed value of the EPSP amplitude; however, the probability of the existence of E to PV connections followed a Bernoulli distribution (Fig. 1C, middle). Therefore, by chance, some PV neurons received more connections from some E neurons within one of the E assemblies. The third network also followed a Bernoulli distribution for the connections from E to PV neurons; however, the EPSP amplitudes were drawn from a log-normal (L-N) distribution (Fig. 1C, bottom), as has been observed experimentally (*29*). This provided the highest level of heterogeneity in E-PV connections among the three networks studied.

We then tracked the IPSPs as they evolved over time under the plasticity rule of Fig. 1B in these three networks. IPSP amplitudes were initialized to be identical in all three networks (Fig. 1D). We observed that the connectivity matrix denoting the IPSP amplitudes from PV neurons to E neurons became structured, for networks with Bernoulli (Fig. 1E, middle) and L-N distributions (Fig. 1E, bottom), but not for the network with FID distribution (Fig. 1E, top). This can be seen by sorting the connectivity matrices such that PV neurons are ordered according to their maximum total IPSP projection onto each excitatory assembly. The networks self-organized such that PV neurons with relatively large values of total summed IPSP amplitude in outgoing connections to one excitatory assembly were more weakly connected, i.e., had smaller total connection strength, to the other excitatory assembly. We then denoted PV neurons with stronger total connection weights (summed IPSPs) to E_1_ (E_2_) as PV_1_ (PV_2_) neurons. The average IPSP amplitude from PV_1_ to E_1_, represented by *w*_*i*1_, grew, and that to E_2_, represented by *w*_*i*2_, decreased as a function of simulation time for networks with both Bernoulli (Fig. 1F, middle; *w*_*i*1_ = −0.400 ± 0.169 mV and *w*_*i*2_ = −0.302 ± 0.152 mV at *t* = 3000 s) and L-N distribution (Fig. 1F, bottom; *w*_*i*1_ = −0.413 ± 0.226 mV and *w*_*i*2_ = −0.277 ± 0.182 mV at *t* = 3000 s). These changes were almost absent for the network with FID distribution (Fig. 1F, top; *w*_*i*1_ = −0.357 ± 0.153 mV and *w*_*i*2_ = −0.345 ± 0.152 mV at *t* = 3000 s). These results indicate that for networks with heterogeneity in E to PV connections, plasticity drove a tendency for individual PV neurons to develop stronger or weaker connections to E neuron populations, but this preference was absent for the network with FID. While for simplicity we considered only PV-to-E plasticity, we confirmed that adding multiplicative Hebbian STDP rules (*30*, *31*) for E-to-PV and E-to-E plasticity results in a similar pattern of IPSP evolution from PV to E neurons (fig. S1).

To determine how this preference emerged, we examined the distributions of the projections from each excitatory assembly onto the PV subnetworks for the different scenarios that we considered. The distribution of the total (summed) EPSP from all E neurons within E_1_ onto individual PV neurons was more distinct and skewed from E_1_ onto PV_1_ than from E_1_ onto PV_2_ (Fig. 1G, middle, bottom) for networks with Bernoulli and L-N distributions. By construction, however, these two distributions were identical for the FID network (Fig. 1G, top).

These results demonstrate that heterogeneity in the existence and the amplitude of the connections from E assemblies to PV neurons can separate the PV population into groups that, by chance, receive more projections from a certain excitatory assembly. Under this form of symmetric PV-to-E plasticity, PV neurons that received stronger total EPSP projections connect to the corresponding excitatory assembly with stronger than average IPSP values, driving population-level reciprocal connectivity between E assemblies and newly emerged PV assemblies. We note that while it was possible that the connectivity could have evolved toward global or cross-group (lateral) inhibition, instead, we find the emergence of a reciprocal pattern of connectivity between PV to E subnetworks. A more systematic analysis of the effect of heterogeneity on the development of feature tuning in PV neurons and their synaptic connections to E neurons is provided in fig. S6.

### Foundations for the emergence of co-tuned PV subnetworks are explained using a mean-field theory

**Fig. 2.**
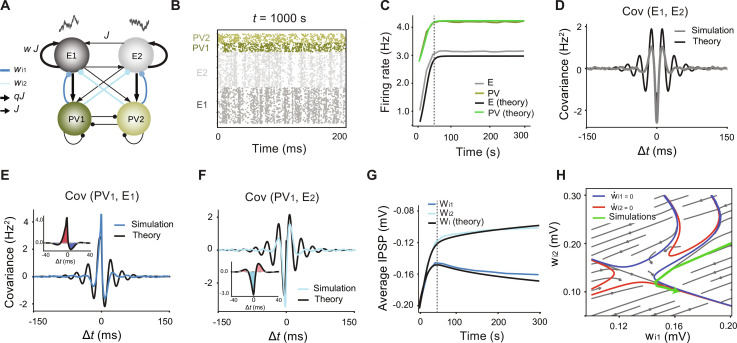
Theoretical understanding of the emergence of co-tuned PV subnetworks. (**A**) Network with two excitatory and two inhibitory populations. The strength of synaptic connections within each excitatory population was *w* = 2.5 times stronger than the PSP amplitudes for the synapses between the excitatory populations. (**B**) Raster plot of the network 1000 s after the onset of the inhibitory plasticity rule. (**C**) Firing rates of the excitatory and inhibitory populations and our theoretical predictions. (**D**) Covariance function between E_1_ and E_2_ average neuronal activity and the theoretical prediction for this function. (**E**) Covariance function between PV_1_ (lead) and E_1_ (lag) and the theoretical match to this function. Inset: The learning signal has a net potentiation effect. (**F**) Covariance function between PV_1_ (lead) and E_2_ (lag) and the theoretical match. Inset: The learning signal has a net depression effect. (D) to (F) were obtained 1000 s after the onset of learning. (**G**) Evolution of the IPSP synaptic values from PV_1_ to E_1_ and E_2_ indicates potentiation and depression, respectively, and our theory captures this phenomenon. Vertical dashed lines in (C) and (G): time point after which the mean population firing rate does not change. (**H**) Vector field of the coupled dynamics between average ${\overset{\cdot }{w}}_{i1}$ and ${\overset{\cdot }{w}}_{i2}$ derived from our theory. The trajectory of these weights obtained from large-scale network simulations (green curve) follows the vector field.

To derive a theoretical understanding of the emergence of tuned PV to E synaptic weights, we constructed a network (Fig. 2A) with two excitatory populations, each receiving 30% of their inputs from a private shared input source (*c* = 0.3). To provide a simplified model of E to PV heterogeneity, we consider from the outset two distinct populations of PV neurons, each receiving stronger input from one of the excitatory assemblies. The connection difference was characterized by a factor *q* > 1 that scaled the stronger EPSP from one of the excitatory populations to a given PV population. Initially, all IPSP amplitudes from the PV populations to the excitatory populations were identical (Fig. 2A). We then used the symmetric inhibitory plasticity rule in Fig. 1B and observed network dynamics and synaptic weight evolutions as a result of this plasticity mechanism. The raster plot of the network 1000 s after the onset of the inhibitory plasticity rule is shown in Fig. 2B.

To explain the interaction between the inhibitory synaptic weight evolution (structural tuning) and the correlation structure between E and PV subnetworks (functional tuning), we developed a mean-field model that derives the joint dynamics of synaptic weights and population-level firing rates (Fig. 2 and Methods). The model self-consistently describes the evolution of both the firing rates and the correlations between firing rates of different neuronal populations as the synaptic weights change over time, and the consequent evolution of the synaptic weights as these firing rates and correlations change over time. This reduced dimensional model helps to reveal how the interaction between the plasticity rule, heterogeneity in the EPSP amplitudes, and the correlation structure between the subnetworks results in the emergence of structurally tuned PV subnetworks.

First, we compare the population firing rates and correlations obtained from the simulations to the predictions from the mean-field model. The model successfully captures the evolution of firing rates (Fig. 2C). It also approximates well the evolution of correlations between the populations (Fig. 2, D to F). We find competition between the E populations (Fig. 2B), characterized by the average population cross-covariance function between the excitatory population firing rates (Fig. 2D). The strong correlations between PV_1_ and E_1_ activity (and similarly between PV_2_ and E_2_), well captured by the mean-field model (Fig. 2, E and F), show a positive covariance between PV_1_ and E_1_, which occurs as a result of the strong EPSP in the connection from E_1_ to PV_1_. However, since E_1_ and E_2_ are negatively correlated, the covariance between PV_1_ and E _2_ is mainly negative around zero-lag intervals (Fig. 2F). This correlation pattern reveals functional co-tuning of PV subnetworks with the E assemblies and results from the initial bias in the E-to-PV connection strength.

Further, the correlation pattern between E and PV subnetworks drives changes in connectivity, or structural tuning, of the network. Given the correlation function between the activity of the presynaptic and a postsynaptic neuron, for any STDP function defining the plasticity rule for synaptic changes, the evolution of the average synaptic weight between the two neurons can be derived using the framework introduced in (*32*). To estimate the average inhibitory synaptic changes, we computed a “learning signal” (see Methods), for which a positive (negative) value indicates synaptic potentiation (depression). Because of the symmetry of the network, the average firing rates of the PV populations are the same. Thus, differences in the inhibitory synaptic weight evolutions arise only from the cross-covariance term. Since the learning signal is positive for *w*_*i*1_ (Fig. 2E, inset), this synaptic weight will grow as a function of the training time. However, the learning signal for the connection between PV_1_ and E_2_ is negative (Fig. 2F, inset), which results in an average depression of *w*_*i*2_ over time. The average inhibitory weights thus separate over time, corresponding to the emergence of co-tuned synaptic weights (Fig. 2G). If instead of the symmetric STDP rule for the inhibitory synapses (Fig. 1B) we had used an asymmetric Hebbian rule, the emerging inhibitory weights would have developed lateral inhibition between the excitatory assemblies, as shown in (*26*).

We can use the Laplace transform to express the system of coupled rate and weight equations in Fig. 2 purely in terms of the synaptic weights (Methods). Plotting the dynamics in the phase plane defined by the average reciprocal (*w*_*i*1_) and lateral (*w*_*i*2_) inhibitory connections (Fig. 2H), we see that the dynamics are characterized by an asymptotic line attractor formed by the intersection of the nullclines of ${\overset{\cdot }{w}}_{i1}$ and ${\overset{\cdot }{w}}_{i2}$ . All initial conditions in the parameter space evolve toward having higher values of *w*_*i*1_ relative to *w*_*i*2_, predicting a robust emergence of tuning. The trajectory that characterizes the evolution of the average inhibitory weights in the large-scale simulations is shown in green on the phase plane (Fig. 2H); its dynamics is well predicted by the system’s flow.

To summarize, our mean-field theory shows how initially identical PV-E connections can self-organize into inhibitory subnetworks with strong reciprocal connections to the E assembly that drives them most strongly. This self-organization is mediated through the symmetric STDP rule and the strong correlations between E and PV subnetworks.

### Internal correlations are necessary to shape inhibitory tuning

**Fig. 3.**
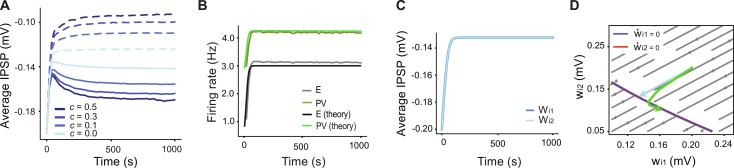
Importance of correlations and consequences of ignoring them. (**A**) Simulation results indicate that increasing the percentage of input correlations c compared to the background input has a marked positive effect on the difference between the IPSP amplitudes to different excitatory assemblies. (**B**) Ignoring the correlation term in the theoretical derivations does not affect the rate dynamics, and our theory can capture the dynamics of the excitatory and inhibitory rates. (**C**) In our theory, the evolution of the IPSP amplitudes from PV_1_ to E_1_ and E_2_ will be identical if the correlation term is removed from the equations. (**D**) Vector field of the system describing w_*i*1_ and w_*i*2_ coupled dynamics (derived from our theory after removing the correlation term between E and PV populations). The nullclines of ${\dot{w}}_{i1}$ and ${\dot{w}}_{i2}$ are exactly equal and their intersection forms a line attractor. The trajectory of the weight evolution from network simulations (green curve) does not match the flow of the vector field (blue trajectory).

While mean-field approaches sometimes neglect the role of correlations, we emphasize that here, they are critical to understanding the observed dynamics. In the emergence of tuned PV to E synaptic weights (structural tuning), two correlation terms play roles. First, E neurons in an assembly receive shared “external” input with a firing rate of *c*η; increasing the value of c increases this correlation term and results in more separation between *w*_*i*1_ and *w*_*i*2_ (Fig. 3A). The second correlation term is generated “internally” between the excitatory and inhibitory neurons, due to E-PV connections with different values of EPSP amplitudes. To demonstrate the importance of these contributions, we compare the results above with those obtained by ignoring all the internal correlations between PV and E neurons for a chosen value of *c* = 0.3 (Eq. 16 in Methods). The steady-state population firing rates evolve similarly to the case with correlations (compare Fig. 3B with Fig. 2C), and simulation and theory converged to similar results. However, one sees a marked difference in the dynamics of the synaptic weight evolution (Fig. 3C): in this case, *w*_*i*1_ and *w*_*i*2_ evolve similarly and, as a result, converge to identical values around the average value of the steady-state weights for the simulation in Fig. 2. This shows a clear mismatch with the simulation results in Fig. 2G. We thus conclude that the internal correlations between the inhibitory and excitatory neurons contribute substantially to shaping tuned PV weights.

In our approach, the vector field of the system without the correlation terms between PV and E neurons has overlaid nullclines for *w*_*i*1_ and *w*_*i*2_, which form a line attractor (Fig. 3D). This line attractor represents *w*_*i*1_ + *w*_*i*2_ = constant, or excitatory firing rate homeostasis. Starting from the same initial weights as in Fig. 2H, under these dynamics, the trajectory moves on a straight line to the closest point on the line attractor (blue trajectory in Fig. 3D), inconsistent with the trajectory from the simulation (green trajectory). Thus, the full mean-field model, including correlations between PV and E neurons, better captures the steady-state values of the inhibitory weights, and its flow field demonstrates the general outcome of tuned PV to E weights.

### Emergence of PV subnetworks generalizes to more realistic networks

**Fig. 4.**
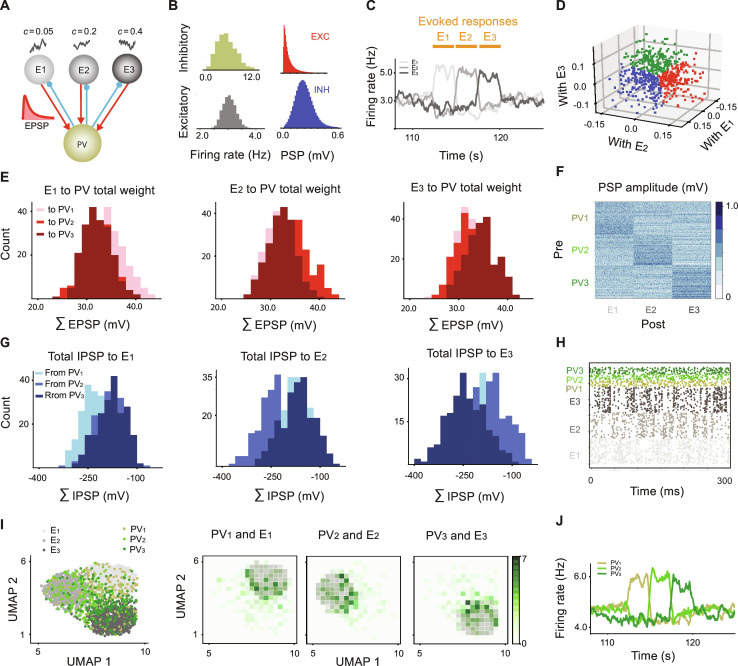
Emergence of tuned PV subnetworks in a more realistic network. (**A**) Network with three excitatory assemblies, each receiving a different level of correlated input, c = 0.05 for E_1_, c = 0.2 for E_2_, and c = 0.4 for E_3_, and initially with a single PV population. All populations are connected to each other, but only reciprocal E-PV connections are shown. (**B**) Left: L-N distribution of E and PV firing rates after plasticity operation for 2000 s. Right: Fixed EPSP amplitudes for the connection weights from E to PV are drawn from an L-N distribution. ∣IPSP∣ amplitudes follow an L-N distribution as a result of plasticity. (**C**) Specific sensory input driving E_1_ (from *t* = 112.5 s to *t* = 115 s), E_2_ (from *t* = 115 s to *t* = 117.5 s), and E_3_ (from *t* = 117.5 s to *t* = 120 s) after plasticity operation. (**D**) Response similarity between individual PV neurons and each assembly E_1_, E_2_, and E_3_ form a three-dimensional cluster of vectors. Blue, red, and green: high similarity with E_1_, E_2_, and E_3_, respectively. (**E**) Total EPSP projections from individual neurons in E assemblies onto the assigned PV populations. (**F**) Connectivity matrix from labeled PV neurons to the excitatory neurons. (**G**) Distribution of the total IPSP amplitudes from individual neurons in each PV population to different E assemblies. (**H**) Raster plot of neuronal activities for the last 300 ms of the spontaneous state. (**I**) Two-dimensional UMAP projection of the neuronal activities during the evoked state (from *t* = 112.5 s to *t* = 120 s). (**J**) E_1_, E_2_, and E_3_ assemblies in the evoked state caused similar evoked responses in PV_1_, PV_2_, and PV_3_ subnetworks, respectively.

To determine whether the emergence we have shown above of functional and structural co-tuning in PV neurons holds for more general and realistic cases, we considered some variations. First, we increased the number of excitatory assemblies and considered networks with three E assemblies. Second, we evaluated the importance of the tuning strength of the E networks. We did this by providing the three excitatory assemblies with different tuning strengths, with each receiving a different degree of private correlated input (Fig. 4A). Finally, for increased generality, we allowed the target firing rates of the excitatory populations to be heterogeneous and drawn from an L-N distribution. As for the system in Fig. 1, we introduced randomness in the E-to-PV connections: The EPSP projection weights followed an L-N distribution (Fig. 4B) to an initially undifferentiated PV population.

As before, we initialized all IPSP projections from the PV to the E assemblies as identical and evaluated the emergence of structurally co-tuned inhibitory connections under the plasticity rule. The evolved distributions of neuronal firing rates for the E and PV populations are shown in Fig. 4B. Given the fixed randomly initialized EPSP synaptic weights, the inhibitory synaptic weights (IPSPs) move toward a heavy-tailed distribution under the symmetric PV-to-E plasticity (Fig. 4B). We then fixed this network and evaluated its functional tuning by injecting a sequence of step sensory inputs to each of the excitatory assemblies in turn (Fig. 4C). Inhibitory neurons did not receive any external sensory input. We then analyzed the network activity, including the “evoked” condition, as well as some “spontaneous” activity before the stimulus, when all populations receive only random inputs. For each PV neuron, one can derive a three-dimensional vector in which each element represents the response similarity between the PV neuron and the average population responses of the three distinct excitatory assemblies (Fig. 4D). We will refer to this measure as the “population similarity measure.” While the responses clearly do not form distinct clusters, we can nonetheless use this measure to assign PV neurons a group identity according to the maximum absolute value of the components of each vector (Fig. 4D), defining populations PV_1_ to PV_3_.

We examined whether using the population similarity measure to assign these labels to PV neurons revealed any specific pattern of connectivity between the E and PV neurons. The clustering of PV neurons, in fact, emerges ab initio from a randomly stronger excitatory drive from distinct excitatory assemblies to a portion of the PV neurons (network heterogeneity in EPSP values). This is reflected in the distribution of the total EPSPs from E_1_ through E_3_ to individual PV populations (Fig. 4E). Under the plasticity rule, we find that this inhomogeneity drives the evolution of tuned PV-E connectivity, shown in the distributions of the total IPSP amplitudes from individual PV neurons to E_1_ through E_3_ (Fig. 4G), where PV_1_ projects strongly onto E_1_ compared to E_2_ and E_3_. This result indicates that clustering PV neurons based on their functional response similarities to the E population activity results in PV labels that reflect reciprocal structural co-tuning (a structured connectivity matrix; Fig. 4F).

If shared correlated input is viewed as a feature that correlates a group of neurons, we can also investigate whether PV neurons developed tuning to those input features. The raster plots of neuronal activities in the spontaneous state showed only weak correlations in activity between PV and E neurons, with an average cosine similarity of 0.00019 (Fig. 4H). However, in the evoked state (Fig. 4C), the response similarity between some of the PV and E neurons increased. This can be shown by applying a two-dimensional Uniform Manifold Approximation and Projection (UMAP) (*33*) to the neuronal activities during the evoked state (Fig. 4I), using cosine similarity measure for distance. While the distribution of the PV neuron responses on the 2D plane was even broader than those of the E neurons, indicating broad tuning of PV neurons in general, PV subnetwork activities formed a denser cluster around the E assemblies to which they were co-tuned. This finding that individual PV neurons are broadly tuned is consistent with most of the experimental reports (*7*–*9*). At the population level, however, PV subnetworks were sharply tuned with their corresponding E assemblies (Fig. 4, C and J). We note that using principal components analysis (PCA) as a measure for dimensionality reduction resulted in similar conclusions (fig. S2). However, as the distances in UMAP are chosen based on cosine similarity between neuronal activities in high dimensions, it is suitable for the purposes of this study.

For the sake of comparison, we used three other measures to assign labels to the PV neurons, i.e., “individual similarity measure” (discussed in the next section), “outgoing PV measure,” and “incoming E measure,” to label PV neurons (for details, see Methods). While there was some variability in the labels assigned to individual PV neurons (fig. S3), all measures resulted in the same conclusions about co-tuning of PV and E neurons (fig. S5).

Together, for more realistic networks with heterogeneities in the target excitatory firing rates, EPSP amplitudes, and levels of shared correlated input, structural and functional co-tuning of PV subnetworks emerges due to the randomness and heterogeneity in the EPSP projecting weights and the symmetric inhibitory plasticity rule, which provides excitatory firing rate homeostasis. We showed that sensory input to individual excitatory assemblies is reflected in the activity of the corresponding PV cluster, indicating the co-tuning of PV neurons at a population level. However, the broader distribution of individual PV neuron responses in the UMAP space indicates the broad tuning of individual PV neurons. Hence, a population measure for feature tuning, such as average population responses to different stimuli, reveals the existence of sharply tuned PV subnetworks. However, the broad tuning of PV neurons shown in UMAP space leads single-neuron measures of co-tuning, such as pairwise response similarity, to also report broad tuning of individual PV neurons. We will further discuss this difference in the following section.

### Co-tuning of PV neurons is a network property that is not reflected in pairwise correlations

**Fig. 5.**
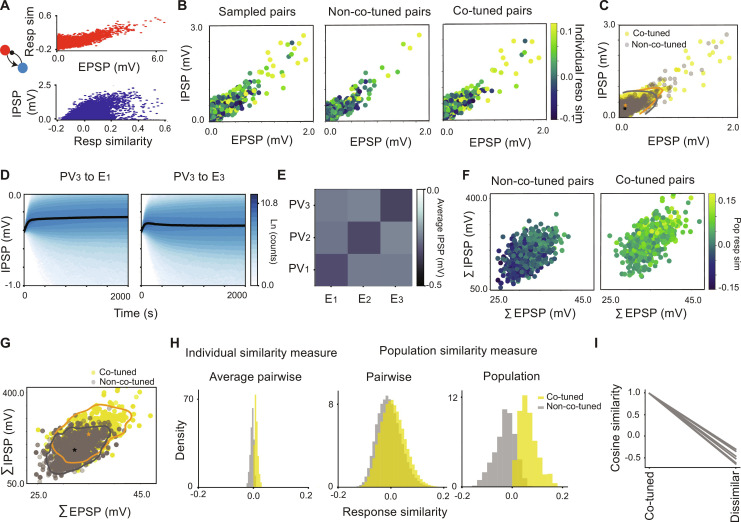
PV tuning as a network property. (**A**) Top: Response similarity as a function of EPSP. Bottom: IPSP as a function of response similarity for reciprocally connected E and PV neurons in the evoked state. (**B**) Left: EPSP and IPSP values for 4000 samples of reciprocally connected pairs. Middle and right: Pairs belonging to non–co-tuned and co-tuned populations. (**C**) Scatterplots of co-tuned and non–co-tuned PSP pairs overlaid on one another. (**D**) Evolution of IPSP distributions from PV_3_ neurons to E_1_ (non–co-tuned) and E_3_ (co-tuned) neurons as a function of time. (**E**) Average connectivity matrix for the synaptic weights from PV subnetworks onto excitatory assemblies. (**F**) Total IPSP projection from individual PV neurons onto co-tuned (right) and non–co-tuned (left) E neurons as a function of the sum of the EPSP. The color bar defines the similarity of the PV neurons with the average excitatory populations. (**G**) Distribution of summed IPSP as a function of summed EPSP for co-tuned (yellow) and non–co-tuned (gray) PV neurons in (H). In (C) and (G), stars indicate the mean of the distributions, and contours define regions in the space that contain 95% of the data from each group. (**H**) Distribution of response similarity between individual PV neurons and E assemblies using the individual similarity measure (left), pairwise (middle), and population (right) response similarities between individual PV neurons and E neurons for co-tuned and non–co-tuned pairs (middle) and populations (right), using the population similarity measure. (**I**) Statistics of cosine similarity between identified PV subnetworks and individual E assemblies in response to the sequential sensory input in (Fig. 4C and J).

There is a close relationship between the functional tuning we discuss above and the structural co-tuning of the PV neurons at the single-neuron level. In the more realistic network introduced in the previous section, we find an emergence of reciprocal structural tuning between E and PV pairs. In the mean-field model, a relatively strong E-to-PV connection weight increases the correlations between E and PV pairs and drives the emergence of functional and structural co-tuning (Fig. 2). In the simulated network, we find that for reciprocally connected E and PV pairs in Fig. 4A, the response similarity between the pairs is correlated with the EPSP size (Fig. 5A). As a result of the symmetric plasticity rule, the IPSP amplitude increases with response similarity (Fig. 5A). Consequently, and similar to the experimental reports in layer 2/3 of mouse visual cortex (*15*, *16*, *18*), we observed a monotonic relation between the EPSP and IPSP amplitudes in all reciprocal motifs in the network under study (Fig. 5B, left). We also note that, in very rare cases (too rare to show up in the random sample shown in Fig. 5B), when the EPSP values are very strong, while the correlation between E and PV pairs increases, the IPSP values start to decrease (fig. S4).

One might expect that E-PV pairs with strong reciprocal synaptic weights and high response similarity belong to co-tuned assemblies, while those with weak correlation coefficients and weak reciprocal synapses belong to non–co-tuned subnetworks. However, this is not the case. The reason is that relatively strong EPSP projections cause strong IPSP, regardless of the identity of the targeting PV neuron. EPSP and IPSP values for pairs that belonged to co-tuned subnetworks (Fig. 5B, right) span the same range as those drawn from non–co-tuned subnetworks (Fig. 5B, middle). Plotting contour lines of the 2D density of the data points for the two groups shows that their synaptic patterns are almost indistinguishable (Fig. 5C, Kullback-Leibler divergence between co-tuned and non–co-tuned EPSP distributions = 0.0265, Kolmogorov-Smirnoff test *P* = 0.478 and Kullback-Leibler divergence between the respective IPSP distributions = 0.283, Kolmogorov-Smirnoff test *P* = 0.0). IPSPs for the PV neuron projections quickly evolve to span a similarly wide range for both co-tuned and non–co-tuned E neurons (Fig. 5D). However, the means of the distributions are different, reflecting the tuning of the subnetworks; the average connectivity matrix from PV to E assemblies, formed by averaging the matrix of Fig. 4F within PV/E blocks, has a diagonal structure that reflects stronger co-tuned synaptic weights from the PV populations to their corresponding co-tuned E assemblies (Fig. 5E). This average also shows that the strength of correlations in the input that formed the different E groups influences the strength of co-tuned weights from the corresponding PV to E groups. The dependence of the inhibitory synaptic strengths on the external correlated input was discussed in Fig. 3A.

While the structural connectivity that corresponds with co-tuning is thus not distinctly apparent in these pairwise measures, it is clearly evident from population measures. If one instead plots the total sum of projecting EPSPs from E assemblies to single PV neurons versus the total IPSP projections from single PV neurons onto the E assemblies, co-tuned and non–co-tuned PV neurons are considerably better separated (Fig. 5F): Higher values of the summed EPSPs on average result in higher summed IPSP values and higher population response similarities between single PV neurons and the entire E populations (Fig. 5F; response similarity with the E populations is shown by the color map). This results in more discriminable distributions for the two groups (Fig. 5G, Kullback-Leibler divergence between the total incoming EPSP distributions for co-tuned and non–co-tuned tuned pairs = 0.345, Kolmogorov-Smirnoff test *P* = 0.0 and Kullback-Leibler divergence between the total outgoing IPSP distributions for co-tuned and non–co-tuned tuned pairs = 0.465, Kolmogorov-Smirnoff test *P* = 0.0). We conclude that to identify structural tuning between subnetworks of neuron types, collective population-level measures, such as the total (or average) coupling PSP amplitudes, result in a more identifiable network structure.

The results presented so far do not depend on the specific measure used to define the PV groups (fig. S5, F and G). For example, one can also define an individual similarity measure in which pairwise response similarities between PV and E neurons are calculated as a three-dimensional vector assigned to each PV neuron, with each component representing the average pairwise similarity with individual neurons in one of the E assemblies. Clustering PV neurons according to this measure results in labeling with 98% overlap with the labels defined above so that identical conclusions follow (fig. S3, C and D). However, to highlight the impact of different measures for PV labeling on conclusions about functional tuning, we compared the distributions of pairwise or population response similarities for the two measures of PV labeling. First, identifying PV groups using individual similarity, we compared the distributions of average individual PV response similarities with individual E neurons in each assembly (Fig. 5H, left). The distributions of co-tuned and non–co-tuned PV neuron average response similarities under this measure were very narrow, with the means of the distributions close to 0 (average response similarity for the co-tuned and non–co-tuned PV neurons: 0.010 and − 0.0048, respectively). Second, we compared the pairwise response similarities between individual PV and E neurons, for co-tuned and non–co-tuned pairs without averaging (Fig. 5H, middle), and we obtained very similar results for both measures, due to the 98% overlap between the PV labels. Third, for the population similarity measure, we calculated the response similarities between individual PV neurons and average E population responses. The distributions of co-tuned and non–co-tuned PV response similarities with each E assembly were more separated (Fig. 5H, right) than those obtained for pairs of PV and E neurons (Fig. 5H, middle). The means of the distributions for co-tuned and non–co-tuned PV neurons were 0.064 and −0.03, respectively, indicating relatively increased response similarity between individual PV neurons and E subnetworks. Finally, we calculated the response similarity between “populations” of PV subnetworks (defined based on the population similarity measure) and E cell assemblies, which resulted in nine different quantities, three of which corresponded to co-tuned subnetwork response similarities (Fig. 5I). The average co-tuned and non–co-tuned response similarities were 0.983 and −0.486, respectively. These quantities improved substantially compared to the previous distributions and reflect highly co-tuned PV subnetworks with E cell assemblies.

These findings demonstrate that in a more realistic network with heterogeneities, there is a monotonic relation between the reciprocal EPSP and IPSP amplitudes for pairs and subnetworks of connected E and PV neurons. However, high values of pairwise response similarity do not necessarily imply that the two neurons belong to functionally co-tuned subnetworks of PV and E neurons. While pairwise measures for tuning may be interpreted as a lack of co-tuning of PV neurons, consistent with previous experimental reports, population measures better reveal structural and functional co-tuning of PV neurons collectively. Average pairwise (individual-individual) similarities are the least sensitive measure with which to define co-tuning of the PV neurons (Fig. 5H, left, middle). Response similarity between individual PV neurons and the entire population (individual-population) is a better representative of co-tuning (Fig. 5H, right), and population-population similarities are the best measures to identify co-tuning of PV neurons with the E assemblies (Fig. 5I).

### Co-tuned PV to E subnetworks reduce feature tuning of the E neurons

**Fig. 6.**
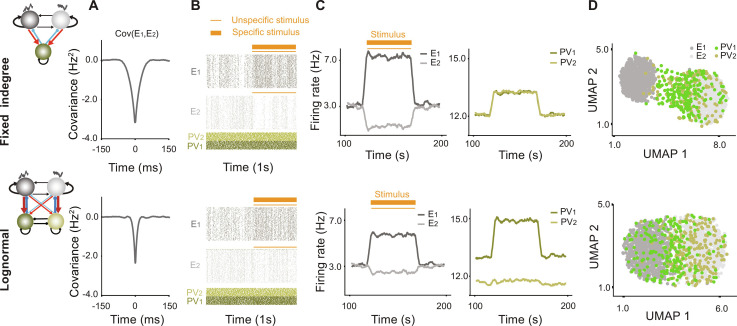
Co-tuned PV subnetworks and feature selectivity of excitatory neurons. (**A**) Cross-covariance between E_1_ and E_2_ dynamics in the spontaneous state for the network with FID (top) and L-N (bottom) distribution for the E-to-PV connections. The synaptic weights for the two networks in Fig. 1 were frozen at *t* = 2000 s. (**B**) Raster plots of the FID and L-N network in the spontaneous and evoked state where E_1_ received a specific sensory input and all E neurons received nonspecific input. (**C**) Average population responses of the E assemblies (left) and PV subnetworks (right) to the sensory input. (**D**) Two-dimensional UMAP projection of the neuronal responses to the sensory input for the FID (top) and L-N (bottom) networks.

How does the emergence of co-tuned PV to E subnetworks affect feature selectivity in the E assemblies? To compare properties of networks with and without co-tuned PV subnetworks, we return to the time-evolved FID and L-N E-PV networks of the first section (Fig. 1). Recall that the FID network did not result in co-tuned PV neurons, while with L-N E-PV weights, the intrinsic heterogeneity of the E-PV connectivity resulted in diversification and co-tuning of the PV population (Fig. 1F, top and bottom). Schematics of the resulting networks are shown in Fig. 6. Intuitively, and as we have previously shown (*26*), an inhibitory architecture that promotes competition between the E assemblies should increase feature selectivity and input amplification in the network. Here, the undifferentiated PV population of the FID network results in more competition between the E assemblies, reflected in more negative cross-covariance between the E populations compared to the L-N network with co-tuned PV populations (Fig. 6A).

To explore the consequences of competition for feature selectivity, we presented an external input specific to E_1_ neurons and a weaker common input to both excitatory assemblies (Fig. 6B). Input amplification of the E_1_ response was stronger for the FID network (Fig. 6C, left), and E_2_ response was more suppressed. In this network, nonspecific increased PV responses suppress the responses of both E assemblies equally (Fig. 6C, right). However, for the L-N network, the PV_1_ response is enhanced due to its correlation with E_1_, prohibiting in turn strong input amplification in E_1_. Further, PV_2_ showed no notable changes in its dynamics and, correspondingly, negligible suppression of the E_2_ response. This implies reduced feature selectivity for the network with co-tuned inhibitory subnetworks.

The reduced feature selectivity in the co-tuned case is evident using low-dimensional UMAP projections of the neuronal responses. For the FID network, as expected, PV responses do not form distinct clusters, and the excitatory clusters are better separated compared with the L-N network, in which E response clusters are closer to each other, indicating reduced feature selectivity. In the L-N network, co-tuning of PV subnetworks is clearly visible. Similar results were obtained using PCA (fig. S2, C and D), showing the generality of our findings regardless of the dimensionality reduction method used.

In general, the larger the variance (or the greater the heterogeneity) of the excitatory connections onto the PV population, given an identical mean for the EPSP projection weights, the more tuned the PV activities become to the excitatory assemblies; this is shown by the examples in Fig. 1 and also a more parametric investigation in fig. S6. This tuning results in amplified PV responses to external sensory input, but comes at the cost of reduced competition between the excitatory assemblies, and therefore less separability and less selective representations in the excitatory neuronal activities.

### Co-tuned E and I subnetworks lead to higher stability and a wider dynamical response range

We now explore the consequences for network dynamics of the existence of such inhibition-mediated E-E competition. We again use the FID and L-N networks of Fig. 1 to investigate the response dynamics and stability of networks with different inhibitory architectures. With all weights frozen following evolution (similar to the previous section), we compared the raster plots, power spectral densities, and the eigenvalue spectrum of the spontaneous activity of these networks for *w* = 2.5, of the previously chosen scalar defining the strength of the excitatory connections within each assembly.

**Fig. 7.**
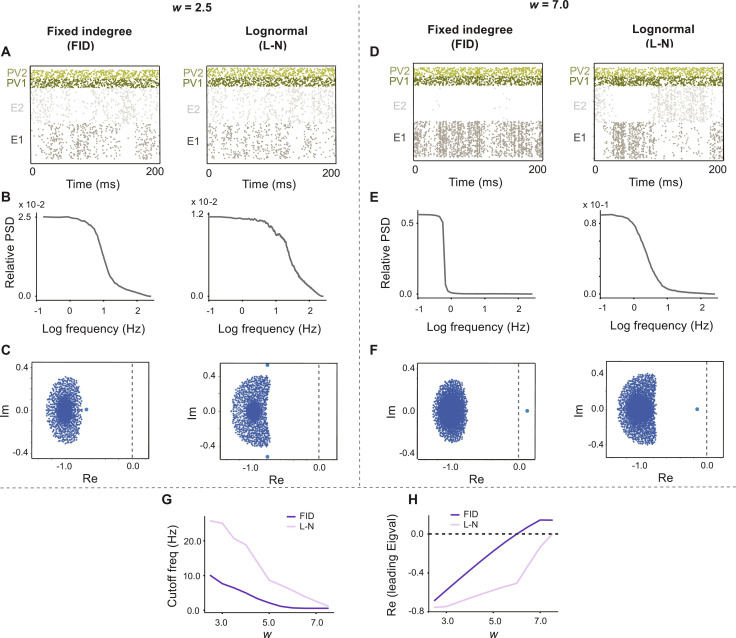
Effect of co-tuned PV subnetworks on network stability and frequency response range. (**A**) Raster plots of the network activity for *w* = 2.5, for the networks with FID and L-N E-to-PV weight distributions, respectively, with frozen weights obtained after 2000 s of training in Fig. 1. (**B**) Power spectral density of the E_1_ firing rate for the FID and L-N network, respectively. (**C**) Eigenvalue spectra of the linearized neuronal dynamics for networks in (A) and (B), respectively. (**D**) Raster plots for stronger within-assembly connections (*w* = 7.0) for networks in (A). (**E**) Power spectral density for the firing rate of E_1_, in the network with FID and L-N distribution, respectively. (**F**) Eigenvalue spectrum of the network dynamics for (*w* = 7.0) for the networks in (D). (**G**) Cut-off frequency as a function of within-assembly coupling strength *w* for the FID and L-N network. (**H**) Real part of the leading eigenvalue for the FID and L-N network as a function of *w*. Dashed black line: Boundary of stability.

Spontaneous activity in the two networks tends to fluctuate between activations of the two E populations. Activity in the FID network shows stronger competition between excitatory assemblies with a relatively longer time of active state for each assembly, while the L-N network shows more rapid switches between high and low activity states for each assembly (Fig. 7A). Correspondingly, the L-N network’s averaged neuronal dynamics for either *E*_1_ or *E*_2_ has a normalized power spectrum exhibiting a higher frequency range than that of the FID network (Fig. 7B), indicating narrower autocorrelation function for the E assemblies (fig. S7). The eigenvalue spectra of both networks, calculated from the Jacobian of the high dimensional system (Fig. 7C), have negative real parts, which indicates that both networks are stable. Note that in both cases, there are two additional complex conjugate eigenvalues on the left side of the bulk, but they are not shown in (Fig. 7C and F).

To test the stability and dynamical ranges of these networks under parameter changes, we increased the value of *w*, and strengthened the connections within each excitatory assembly (*w* = 7.0 here), leaving PV to E connections as they were. This simulates a condition under which E connections change rapidly due to learning, but inhibitory plasticity is much slower (*34*). Under this condition, the E populations show stronger degrees of synchrony (Fig. 7D). The L-N network still exhibits faster dynamics than those of the FID network (Fig. 7E). However, at this connectivity strength, the FID network now has a leading eigenvalue that has crossed the edge of stability at zero (Fig. 7F), indicating unstable dynamics (corresponding to synchronous activity). In contrast, all eigenvalues of the L-N network, including the leading eigenvalue, lie on the left side of the stability boundary, and therefore, the network remains stable (Fig. 7F). The leading eigenvalue for the L-N network is less than the FID network across a wide range of values of w (Fig. 7G), and the bandwidth is also wider (Fig. 7H). These results suggest that if connection strengths within excitatory assemblies increase, a network with co-tuned PV subnetworks will be better able to maintain the stability of the network.

## DISCUSSION

In this study, we first showed that inhibitory tuning can emerge from randomness in network architecture. Our examples and mean-field analysis show how PV neurons can develop structural and functional co-tuning with E neurons by considering the “network effects” of heterogeneity in the EPSP connections from E to PV neurons (*8*, *12*, *22*), correlated shared input from other layers (*20*), and a symmetric STDP rule governing the synaptic changes from PV to E neurons (*23*, *24*, *26*, *28*). Our results suggest that the integration of random local excitatory activity by PV neurons (*7*), combined with correlation patterns of excitatory assemblies and the heterogeneity of the EPSP amplitude, can explain the co-tuning of PV with E neurons, reported in mouse cortex (*14*–*16*, *19*) [also see (*35*, *36*)] as well as the general co-tuning of excitation and inhibition in the zebrafish homolog of the olfactory cortex (*37*). These assumptions can also explain the monotonic relationship between EPSP and IPSP in reciprocally connected E and PV neurons (*15*, *16*).

Second, as our theoretical and simulation results indicated that population-level heterogeneity and randomness alone can cause PV (co-)tuning, we conjectured that population measures can better capture this tuning. We demonstrated how weakly tuned individual PV neurons can develop sharp co-tuned subnetworks with the E assemblies by highlighting the difference between individual and population-level measures for tuning. More specifically, at the pairwise level, for a network simulation with heterogeneous parameters, highly correlated pairs with strong reciprocal EPSP and IPSP were found in both co-tuned and non–co-tuned E and PV assemblies (Fig. 5B). Therefore, pairwise response similarity between E and PV neurons may not provide a good measure to determine the co-tuning of the PV neurons. In contrast, population measures such as population response similarity result in well-separated distributions for co-tuned and non–co-tuned PV neurons (Fig. 5F). Further, similarities between population responses of PV and E neurons to input features notably increase when the entire subnetwork response, but not single-neuron responses, is considered (Figs. 4I and 5H compared with Figs. 4, C and J, and 5I). Thus, we hypothesize that reports of lack of tuning in inhibitory neurons might be driven by the use of single-neuron measures (i.e., single-neuron responses to visual stimuli) used in those studies (*7*–*11*). Indeed, due to integration from a large pool of differently tuned E neurons, correlations (similarities) between PV and E neurons are weak. However, population-level correlations are high due to the collective structural co-tuning of the PV and E neurons. This phenomenon follows the same principle as described in balanced random networks [weak pairwise correlation between E and I neurons, but high correlation between I and E populations (*38*, *39*)]. Here, we showed how subnetworks of PV neurons develop co-tuning with subnetworks of E neurons due to the overall randomness and heterogeneity in the connectivity from E to PV neurons, in combination with correlated shared input, defining feature tuning for the E assemblies. This is consistent with the idea of pooling (*7*) if PV correlations are measured with E subnetworks rather than single E neuron activities (Fig. 5I).

One experimental prediction of our theoretical study is that population-level measures can reveal functional and structural tuning of PV neurons not evident in single-neuron measures. For example, given our findings in Fig. 4, I and J, in experiments with simultaneous recordings from PV and E neurons, one could look for the existence of groups of PV neurons that on the population level are highly correlated with groups of E neurons, and check if pairwise correlations between E and PV neurons reflect this functional co-tuning. Our theory suggests that even with weak pairwise PV-E correlations, it is possible to get highly co-tuned PV and E subnetworks. Further, to test model predictions about the structural tuning of PV neurons, one could test in electron microscopy (EM) data whether, when defining a group of E neurons by the existence of projections onto a set of PV neurons, there are enhanced reciprocal connections in return from that PV group to the E neurons. Ideally, this would be combined with analysis of functional data that would elucidate whether co-tuning of these groups exists (*40*, *41*).

We showed that heterogeneity in the connections from excitatory to inhibitory neurons was an important component in our model to get functional and structural co-tuning of inhibitory neurons. In the cortex, considering the spatial dependence of EPSP amplitudes (*16*, *42*, *43*), PV neurons that are located more closely to a group of E neurons receive more excitatory inputs from those neurons. This reflects the skewed randomness and heterogeneity in the EPSP connections that we used in our paper. The emerging structurally co-tuned subnetworks provide a detailed balance between excitatory and inhibitory neurons, as shown in a network with feedforward inhibition and at the single-neuron level in (*25*). Therefore, neurons are maintained in the balanced state over time (*38*, *44*–*46*) and over space (*18*). This can be important in stabilizing spatiotemporal patterns in the cortex. It was shown that considering the spatial profile of recurrent connectivity in a network model with different inhibitory subtypes could capture the tuning properties of different inhibitory subtypes and resulted in a better agreement with the large-scale recordings in mouse V1 (*47*).

The second important component of our model was the homeostatic plasticity for the inhibitory to excitatory weights. Other STDP mechanisms, including asymmetric STDP functions, have been reported for inhibitory synapses in different parts of the brain (*26*, *28*, *48*, *49*). For example, it has been shown that in the orbitofrontal cortex of mice, somatostatin (SOM) neurons follow an asymmetric Hebbian rule for their synapses onto excitatory neurons (*26*), and that, using the methods described here, this STDP rule results in the emergence of lateral inhibition from SOM neurons onto excitatory subnetworks (*26*). Our mean-field theory can capture the evolution of inhibitory synaptic weights and accordingly predict the emerging network structure for any shape of STDP curves and average pairwise correlations based on the formalism developed in (*32*).

The third component that we used in our model was shared correlated input from layer 4 impinging on different subnetworks of excitatory neurons. In our model, while both excitatory and inhibitory neurons received a background input from layer 4, we considered separate sources of shared correlated input, which defined tuning for individual E assemblies. It is known that shared input from cortical layer 4 and also within layer 2/3 forms fine-scale subnetworks of excitatory neurons in layer 2/3 (*20*) with stronger connections between them (*27*). These strong E-to-E connections play a role in shaping stimulus-specific feature selectivity (*21*). In mouse V1, it was shown that the pattern of correlation between excitatory neurons is strongly influenced by the external stimuli, emphasizing the importance of feedforward connections in shaping responses among E neurons. However, correlation patterns among PV neurons are much less affected by the external input but rather are shaped by the local projections from E neurons within layer 2/3, reflecting the importance of recurrent connections in driving response patterns of PV neurons (*8*). We used this evidence in our computational study and showed that excitatory assemblies formed via shared correlated input could drive the responses of PV neurons through recurrent connections and form co-tuned subnetworks. Had we considered the same layer 4 shared correlated inputs to PV neurons, due to the externally imposed correlated pattern between E and PV neurons, we would have more easily obtained the same results as we have shown here.

Using mean-field theory, we demonstrated that correlations between E and PV neurons played an essential role in shaping tuned connections from PV to E neurons, resulting in a largely monotonic relationship between EPSP and IPSP for the reciprocal connections when a symmetric STDP rule was used. Ignoring the correlation between E and PV populations did not capture the tuned weights observed in the simulations (Fig. 3), likely accounting for recent results in which the EPSP/IPSP relationship did not arise only from heterogeneity (*50*); in that case, the correlation term was ignored. Here, we showed that correlations between the activities of the E and PV neurons were sufficient to get reciprocal and monotonic EPSP and IPSP weights as observed experimentally (*51*). These correlations, at both the pairwise and population level, link functional co-tuning of PV to E neurons to their structural tuning.

Simplified models such as we have proposed are helpful, as they are intended to isolate and study the minimal circuits required to provide a simple mechanistic understanding of the brain. However, to make sure that certain assumptions in our model did not bias our understanding of the phenomenon we reported, we considered different network parameters and different variations: We used current-based leaky integrate-and-fire (LIF) models with delta (for the main paper and our mean-field theory) and alpha synapse models (fig. S1), networks composed of two, three (in the main paper), and four (fig. S6) subnetworks of E neurons, with either only inhibitory plasticity or combined excitatory and inhibitory plasticity (fig. S1), different distributions for heterogeneity (Fig. 1 and fig. S6) and probability of connections (fig. S1), randomness in the target excitatory firing rates (Fig. 4), different correlation levels for the external input (Fig. 3A), and different individual- or population-level measures to assign labels to PV neurons (Fig. 4 and fig. S6). For all these different parameter sets and variations, we observed emerging functional and structural tuning of PV neurons. This indicates that the basic requirements for our model, mainly heterogeneity in the EPSP projections to the PV neurons, homeostasis, and external and internal E-PV correlations, are necessary components for the emergence of PV co-tuning with excitatory neurons.

It is known that the (visual) cortex is composed more of excitatory neurons than inhibitory neurons (*52*, *53*); however, recurrent IPSPs are stronger than recurrent EPSPs to maintain a stable, balanced state in the existence of external input (*39*). We incorporated this fact into our model. More excitatory than inhibitory neurons could potentially intensify the role of E-to-PV heterogeneity in shaping PV functional tuning because of randomness and diverse projections onto the PV neurons. Also, due to the smaller number of inhibitory neurons than excitatory neurons in our model, it is expected that during homeostasis, cooperativity between the small number of inhibitory neurons to regulate the firing of bigger excitatory populations increases. Such an elevated synergy between IPSP amplitudes of a subset of inhibitory neurons that are functionally tuned to a subnetwork of excitatory neurons could enhance the emerging structural tuning of the PV subnetworks in our model. In sum, having more E neurons than PV neurons favors the emergence of both functional and structural PV tuning.

The emergence of structurally co-tuned PV subnetworks with excitatory assemblies can profoundly shape neuronal dynamics. We showed a direct relationship between competition between E assemblies and their feature tuning and input amplification. Competition and input amplification are compromised in networks composed of co-tuned inhibitory subnetworks due to the tight tracking of excitatory activities by the PV subnetworks. It was shown that in balanced networks, the difference between the local excitatory and inhibitory activity is amplified by the network and generates fluctuations in the recurrent circuit self-consistently (*54*). Therefore, the structure of inhibition can have an important role in shaping the fluctuations of the E neurons’ activity. The difference between the excitatory and inhibitory firing rate fluctuations in a network with specific inhibitory subnetworks is smaller than those in a network with one unspecific inhibitory population. Therefore, the amplification of fluctuations in the spontaneous state (and, equivalently, the external input) for the former network is weaker. However, for a network with one global inhibitory population, the inhibitory firing rate is almost constant, providing global balance and keeping a relatively fixed sum of all the excitatory firing rates in all assemblies combined, with relatively little fluctuation in the inhibitory dynamics (*55*). This can result in an attractor network that is mediated by uniform inhibition (*55*, *56*). In our study, we assumed distinct and well-separated excitatory cell assemblies, however, there might be no clear distinction between the excitatory assemblies encoding features with high degree of overlap. In that case, the co-tuning of inhibitory subnetworks, as shown in Fig. 4J, will lose salience.

For feedforward networks with homeostatic inhibitory plasticity, it has similarly been shown that increased network stability results in decreased feature selectivity (*25*). Here, we showed that in a recurrent network, reduced selectivity and input amplification in reciprocally connected E and PV subnetworks come as costs for providing circuit stability (*26*), as shown in the eigenvalue spectrum, and increased dynamical range of the excitatory neuron activity. Since the reciprocal PV to E connectivity structure expands the frequency response of the spontaneous activity (*37*, *57*), it can also play a role in shaping network oscillations. It has been observed that inhibiting PV neurons results in a suppression of gamma oscillations in vivo (*58*, *59*), while their activation stimulates gamma oscillations (*58*). Both gamma oscillations (*58*) and strong reciprocal excitatory to inhibitory connections (*60*) have been shown to enhance signal propagation and reduce noise in the network. The theory developed in our study predicts that the reciprocal connections are responsible for this noise attenuation.

Here, we only addressed the role of a single inhibitory cell type, i.e., PV neurons. It has been reported that SOM neurons are tuned to specific excitatory populations (*61*) and may also play a role in shaping PV tuning through modulation of correlations. It was shown that random projections from SOM to PV neurons could, by chance, target a group of PV neurons more strongly than other groups, and this could also result in tuned PV to E connections mediated through SOM neurons (*26*). Additionally, SOM neurons participate in a disinhibitory circuit through the connections from vasoactive intestinal polypeptide expressing (VIP) to E neurons and may paint a more complicated picture when considered in this analysis (*26*, *62*). It would be interesting to study the complementary role of VIP and SOM in shaping the feature specificity of PV neurons in more detail, and study the interplay between functional and structural properties of networks with multiple inhibitory cell types.

Finally, here, we described how functional network properties, such as correlations between E and PV neurons, shape the network structure (such as the emergence of structurally co-tuned inhibitory subnetworks). We also demonstrated how this emerging structure shapes the functional properties of the network (such as increased network stability at the cost of reduced feature selectivity). Hence, our work describes how network structure and function affect one another self-consistently. This can bring us a step closer to a mechanistic understanding of the dynamic brain.

## METHODS

### Network models

Network simulations were conducted in NEST (*63*), using LIF neuronal dynamics with current-based synapses with delta functions. In all simulations, PV neurons comprised 20% of the network size and the rest of the network was composed of excitatory neurons. In our study, we considered different networks with different sizes and parameters.

For all simulations, the dynamics of the membrane potential for each neuron follows

$$\tau \overset{\cdot }{v}=-v+\mu +\sigma \sqrt{\tau }\xi (t)$$ (1)

in which μ and σ are the mean and standard deviations (SDs) of the total (external plus recurrent) input to the neuron, and are obtained from the following equations using mean-field theory

$$\begin{array}{c} \mu =\sum_{j=1}^{n}\tau N_{j}R_{j}P_{j}W_{j}+\tau \sigma_{i}^{2}J\\ \sigma^{2}=\sum_{j=1}^{n}\tau N_{j}R_{j}P_{j}{W^{2}}_{j}+\tau \sigma_{i}^{2}J^{2} \end{array}$$ (2)

in which τ is the time constant of the membrane potential, *n* is the number of excitatory and inhibitory populations, *N_j_* reflects the number of neurons within each presynaptic population, and *R_j_* represents the average firing rate per neuron for each population, which should be solved self-consistently and follows *f*(μ, σ) in Eq. 6. The variable *P_j_* is the probability of connections from the presynaptic neuron to the postsynaptic neuron, and *W_j_* is the average signed PSP amplitude for the connection weights from the presynaptic to the postsynaptic neuron. The second term in both equations represent the influence of the external input on the dynamics of the membrane potential in which σ*_i_* is the SD of the external Poisson input and *J* is the EPSP amplitude for the connections from the external source to the neuron. Because of the central limit theorem, for a neuron embedded in the network in the asynchronous irregular state, one can assume that the input each neuron receives is close to a Gaussian process. Hence, ξ(*t*) in Eq. 1 is taken to be a Gaussian process with zero mean and unit SD. When the membrane potential reaches the spiking threshold level (defined by the variable θ in table S1), the neuron fires and sends a spike to all postsynaptic neurons.

### Inhibitory plasticity

In all network studies reported in the main article, only the synapses from PV to E neurons were plastic and were governed by a symmetric STDP function (*25*) as follows

$${\mathrm{pv}}_{\text{stdp}}\left(t\right)=\text{exp}\left(\frac{-|t|}{\tau_{\text{stdp}}}\right)$$ (3)

The variable *t* represents the time interval between the pre- and postsynaptic spikes, and as opposed to Hebbian learning, the causal relation between them does not affect the synaptic weight update. Spike emission by the presynaptic PV neuron causes a depression of the synapse. These conditions were shown to be required for the plasticity rule to ensure postsynaptic firing rate homeostasis (*25*).

The weight dynamics from PV to E neurons using the homeostatic plasticity rule in (*25*) are composed of two parts, as follows

$$\begin{array}{c} \overset{\cdot }{w}\left(t\right)=\int_{-\infty }^{+\infty }\mathrm{pv}\left(\hat{t}\right).\text{Cov}\left[r_{e}\left(t\right),r_{i}\left(t+\hat{t}\right)\right]d\hat{t}\\ +\int_{-\infty }^{+\infty }\mathrm{pv}\left(\hat{t}\right).\left(r_{e}^{*}-r_{e}^{\text{target}}\right)r_{i}^{*}\;d\hat{t} \end{array}$$ (4)

The first term on the right-hand side of the equation above reflects the contribution of the cross-covariance function between the pre- and postsynaptic neuronal activities, represented by $\text{Cov}\left[r_{e}\left(t\right),r_{i}\left(t+\hat{t}\right)\right]$ , in which *r*_e_(*t*) and *r*_i_(*t*) represent the temporal fluctuations around the piecewise linear mean firing rates of the excitatory and PV neurons. This covariance term is calculated based on Eqs. 12 and 14, in the time domain and Laplace domain, respectively. We call the first term on the right-hand side of Eq. 4 the learning signal, which drives growth and decay of some inhibitory weights, especially after firing rate convergence. The second term represents the effect of mean firing rates ( $r_{e}^{*}$ for the excitatory postsynaptic neuron and $r_{i}^{*}$ for the inhibitory presynaptic PV neuron). Transient firing rates converged to stationary solutions within the first few 10 s of the simulations in most of our network studies (Fig. 2C). However, given a constant stationary background input, most PV to E weight changes happened after this settlement to the stationary mean solution. A mean-field analysis to explain the emergence of PV weight tuning on the network level is provided in the following sections.

To demonstrate that our results are not affected when excitatory weights also obey a standard plasticity rule, such as Hebbian STDP, we included E-to-E Hebbian plasticity, as well as E-to-PV Hebbian plasticity, in simulations shown in fig. S1. In the main paper, we used 40% probability of connections between PV-PV and E-PV neurons. For the simulations in fig. S1, we also used high connection probabilities (80%) for PV-PV, E-PV, and PV-E synapses, consistent with experimental data (*22*). Moreover, we chose a target firing rate of 1 Hz for the excitatory neurons, while in the main paper those target rates were chosen to be equal to 3 Hz. We note also that in our mean-field theory and simulations in the main paper, we considered delta synaptic dynamics. In fig. S1, we used alpha synaptic dynamics for all connections and showed that the generality of our results is not affected by these changes.

### Response similarity

To calculate the response similarity between PV and E neurons, we considered individual instantaneous neuronal firing rates as vectors in a high dimensional space and calculated the cosine of the angle between the vectors. In other words, we used the following formula

$$\text{Response similarity}=\frac{\left(r_{1}-{\bar{r}}_{1}\right)\cdot \left(r_{2}-{\bar{r}}_{2}\right)}{\;\left\|r_{1}-{\bar{r}}_{1}\right\|\;\left\|r_{2}-{\bar{r}}_{2}\right\|\;}$$ (5)

where *r*_1_ and *r*_2_ are the firing rates of the presynaptic and postsynaptic neurons, the operator in the numerator is an inner product, and ∥.∥ is the Euclidean norm of the responses for each neuron.

### Measures used to label PV neurons

Apart from the population similarity measure explained in the main text, we used three other measures to assign labels to PV neurons. Among those, two of them were network measures: The outgoing PV measure, where the sum of the outgoing connections from each PV neuron to the neurons in the three excitatory assemblies was measured and the maximum projection weight among the three vector components defined the label for individual PV neurons (figs. S3 and S5). The incoming E measure, which calculates the total EPSP projection weights onto each PV neuron and the label of the PV neuron, is defined based on the maximum incoming weight from each E assembly. We also used a measure based on pairwise neuronal response similarities, namely, individual similarity measure, where the average response similarity between pairs of PV and E neurons was used to label PV neurons. The intersection of PV labels for all these four measures are shown in fig. S3, C and D, indicating that individual and population similarity measures result in the highest overlap between PV labels, but in general, all measures have a substantial overlap between the labels.

### Dimensionality reduction method

To reduce the high dimensional activities of the neurons, we used UMAP for dimension reduction (*33*) in the main paper, and we used the cosine measure to identify the distance between data points for the reduced dimensional representation. This method uses algebraic topology to preserve more of the global structure of the data, compared to other dimension reduction methods. We compared the results with another method for dimensionality reduction, namely, PCA, in fig. S2 and obtained similar conclusions.

For the network simulations in Figs. 4 and 5, weak sensory inputs (2% of the total background input for the shared external input, and 2% of the total background input for the external uncorrelated input to the E neurons) were presented to *E*_1_, *E*_2_, and *E*_3_ populations at *t* = 112.5 s, *t* = 115 s, and *t* = 117.5 s, respectively, with frozen weights, obtained 2000 s after the onset of the inhibitory STDP rule. Labeling of the PV neurons was based on their response similarity to the excitatory population responses to these external sensory inputs (population response similarity measure). Instantaneous firing rates of all neurons were calculated with a temporal resolution of 15 ms, for a total duration of 7.5 s (from *t* = 112.5 s to *t* = 120 s). To show the response similarity of the PV and E neurons, we subtracted the mean of the neuronal responses. These neuronal fluctuations were used for the UMAP plots in Fig. 4. Results shown in Fig. 6D were obtained similarly for neuronal responses between *t* = 100 s and *t* = 200 s.

### Mean-field theory for the emergence of PV structural tuning

We study the interactions between the dynamics of LIF neurons and the evolution of plastic weights between PV neurons and excitatory neurons in different populations. We used mean-field theory to investigate how inhibitory weights self-organize relative to the excitatory populations.

The membrane potential for a LIF neuron that is isolated from the network is a low-pass filter of first order with the dynamics represented by *v* in Eq. 1, where μ and σ are the mean and SD of the input signal and ξ(*t*) is assumed to be a zero-mean normal Gaussian white noise. Upon crossing the threshold θ, the membrane potential resets to the level defined by *v*_reset_. When this neuron is embedded in a network, the mean and SD of the total input (external plus recurrent input) have to be calculated self-consistently (*39*, *64*).

The firing of excitatory neurons is correlated, representing feature tuning, via a source of shared input, which projects identical spike trains to all excitatory neurons with equal weight. While inhibitory neurons receive independent background Poisson input with an SD of $\sigma_{i}\sqrt{\tau }\xi \left(t\right)$ affecting their membrane potential, excitatory neurons receive inputs from two sources: a shared Poisson source that is common between E neurons within an assembly and has an SD equal to $\sqrt{c\tau }\sigma_{i}\xi_{c_{1}}\left(t\right)$ , and a background-independent source with an SD of $\sqrt{\left(1-c\right)\tau }\sigma_{i}\xi \left(t\right)$ . The total variance of the entire external input impinging on E neurons is equal to that received by the PV neurons from the background input.

To get neuronal correlation functions, it is required to linearize the dynamics of individual neurons around their operating stationary firing rates. A transfer function that relates the stationary input to the firing rate of the neuron is obtained from the stationary solution of the Fokker-Planck equation, which solves a first passage time problem. The stationary firing rate *r* is obtained from *r* = *f*(μ, σ), where the transfer function *f*(.) follows (*64*)

$$\begin{array}{c} f^{-1}\left(\mu ,\sigma \right)=\tau_{\text{ref}}+\tau \sqrt{\pi }\int_{\frac{v_{r}-\mu }{\sigma }}^{\frac{\theta -\mu }{\sigma }}e^{u^{2}}\left[1+\text{erf}\left(u\right)\right]\;du\\ =\tau_{\text{ref}}+\tau \sqrt{\pi }\int_{y_{r}}^{y_{\theta }}G\left(u\right)\;du \end{array}$$ (6)

To linearize the transfer function around the operating point r*, we take the derivative of both sides with respect to μ. This results in

$$\frac{\partial f}{\partial \mu }=f^{2}\left(\mu ,\sigma \right)\left(\frac{\tau \sqrt{\pi }}{\sigma }\right)\left[G\left(y_{\theta }\right)-G\left(y_{r}\right)\right]=k\left(\mu ,\sigma \right)$$ (7)

where *k*(μ, σ) represents the slope of the *f-I* curve at the linearization point, and $y_{r}=\frac{v_{r}-\mu }{\sigma }$ and $y_{\theta }=\frac{\theta -\mu }{\sigma }$.

Here, *E*_1_ and *E*_2_ represent the average fluctuations of individual firing rates in the E assemblies, and *I*_1_ and *I*_2_ represent the dynamic firing rates of PV neurons. In our model, a stronger connection weight from *E*_1_ to *I*_1_ compared to *I*_2_ differentiates *I*_1_ from *I*_2_; similarly stronger connections exist from *E*_2_ onto *I*_2_ neurons.

The operating points for E and I neurons are r*_e_ and $r_{i}^{*}$ , respectively, where the stationary solution comes from the solution of coupled Fokker-Planck equations. The size of the E population is *N_e_* neurons, and that of the I population is *N_i_*. The probability of connection between E neurons is *P* = 0.1, and every other probability of connection is 4*P* = 0.4 (hence the factors of 4 in Eq. 8). The connection weight from E population *j* to E population *i* is *w_ij_*. Two excitatory neurons residing in different excitatory assemblies, if connected, have a PSP amplitude of *J* mV. However, neurons within each excitatory assembly are more strongly connected (PSP amplitude of *w J*, where *w* > 1). Projection weights from excitatory onto co-tuned inhibitory neurons are equal to *q J*, with *q* > 1; however, projection weights from excitatory to non–co-tuned inhibitory neurons are equal to *J*. The average connection weight from a PV neuron onto an excitatory neuron in assembly *k* is *w_ik_*. The fixed (non-plastic) inhibitory weights between inhibitory neurons are −*gJ*, where *g* = 10. All inhibitory weights from *I*_1_ and *I*_2_ to all excitatory neurons are initially equal to –*gJ*; however, inhibitory plasticity will change these weights, and we are interested in the dynamics of those changes.

The average dynamic mean-field equations for individual neurons around their steady-state values are

$$\begin{array}{c} \tau {\overset{\cdot }{E}}_{1}=-E_{1}+\tau {\mathrm{pk}}_{e}\left(\mu ,\sigma \right)\left(N_{e}{\mathrm{wJE}}_{1}+N_{e}{\mathrm{JE}}_{2}-4N_{i}w_{i1}I_{1}-4N_{i}w_{i2}I_{2}\right)\\ +k_{e}\left(\mu ,\sigma \right)\left[\sqrt{c\tau }\sigma_{i}\xi_{c_{1}}\left(t\right)+\sqrt{\left(1-c\right)\tau }\sigma_{i}\xi \left(t\right)\right]\\ \tau {\overset{\cdot }{E}}_{2}=-E_{2}+\tau {\mathrm{pk}}_{e}\left(\mu ,\sigma \right)\left(N_{e}{\mathrm{JE}}_{1}+N_{e}{\mathrm{wJE}}_{2}-4N_{i}w_{i2}I_{1}-4N_{i}w_{i1}I_{2}\right)\\ +k_{e}\left(\mu ,\sigma \right)\left[\sqrt{c\tau }\sigma_{i}\xi_{c_{2}}\left(t\right)+\sqrt{\left(1-c\right)\tau }\sigma_{i}\xi \left(t\right)\right]\\ \tau {\overset{\cdot }{I}}_{1}=-I_{1}+\tau {\mathrm{pk}}_{i}\left(\mu ,\sigma \right)\left(4{\mathrm{qN}}_{e}{\mathrm{JE}}_{1}+4N_{e}{\mathrm{JE}}_{2}-4N_{i}{\mathrm{gJI}}_{1}-4N_{i}{\mathrm{gJI}}_{2}\right)\\ +k_{i}\left(\mu ,\sigma \right)\left[\sqrt{\tau }\sigma_{i}\xi \left(t\right)\right]\\ \tau {\overset{\cdot }{I}}_{2}=-I_{2}+\tau {\mathrm{pk}}_{i}\left(\mu ,\sigma \right)\left({\mathrm{qN}}_{e}{\mathrm{JE}}_{1}+4{\mathrm{qN}}_{e}{\mathrm{JE}}_{2}-4N_{i}{\mathrm{gJI}}_{1}-4N_{i}{\mathrm{gJI}}_{2}\right)\\ +k_{i}\left(\mu ,\sigma \right)\left[\sqrt{\tau }\sigma_{i}\xi \left(t\right)\right] \end{array}$$ (8)

which in matrix form can be represented as

$$\begin{array}{c} \left(\begin{array}{c} {\overset{\cdot }{E}}_{1}\\ {\overset{\cdot }{E}}_{2}\\ {\overset{\cdot }{I}}_{1}\\ {\overset{\cdot }{I}}_{2} \end{array}\right)=-\frac{1}{\tau }\left(\begin{array}{c} E_{1}\\ E_{2}\\ I_{1}\\ I_{2} \end{array}\right)+p\left(\begin{array}{cccc} k_{e}N_{e}\mathrm{wJ} & k_{e}N_{e}J & -4k_{e}N_{i}w_{i1} & -4k_{e}N_{i}w_{i2}\\ k_{e}N_{e}J & k_{e}N_{e}\mathrm{wJ} & -4k_{e}N_{i}w_{i2} & -4k_{e}N_{i}w_{i1}\\ 4{\mathrm{qk}}_{i}N_{e}J & 4k_{i}N_{e}J & -4k_{i}N_{i}\mathrm{gJ} & -4k_{i}N_{i}\mathrm{gJ}\\ 4k_{i}N_{e}J & 4{\mathrm{qk}}_{i}N_{e}J & -4k_{i}N_{i}\mathrm{gJ} & -4k_{i}N_{i}\mathrm{gJ} \end{array}\right)\\ \left(\begin{array}{c} E_{1}\\ E_{2}\\ I_{1}\\ I_{2} \end{array}\right)+\left(\begin{array}{ccccc} k_{e}\sqrt{c/\tau }\sigma_{i} & 0 & 0 & 0 & k_{e}\sqrt{\left(1-c\right)/\tau }\sigma_{i}\\ 0 & k_{e}\sqrt{c/\tau }\sigma_{i} & 0 & 0 & k_{e}\sqrt{\left(1-c\right)/\tau }\sigma_{i}\\ 0 & 0 & 0 & 0 & k_{i}\sqrt{1/\tau }\sigma_{i}\\ 0 & 0 & 0 & 0 & k_{i}\sqrt{1/\tau }\sigma_{i} \end{array}\right)\;\left(\begin{array}{c} \xi_{c_{1}}\\ \xi_{c_{2}}\\ 0\\ 0\\ \xi \end{array}\right) \end{array}$$ (9)

Note that σ appearing in the slope of the *f*-*I* curve is different from σ*_i_*, the SD of the external inputs; rather, it has two components, which include σ*_i_* and also the internally generated SD (*64*). The fluctuation of the rate dynamics around the fixed point can be written in the following general form

$$\begin{array}{c} \overset{\cdot }{X}=\mathrm{AX}+B\Xi \\ X=\left(\begin{array}{c} E_{1}\\ E_{2}\\ I_{1}\\ I_{2} \end{array}\right)\\ A=p\left(\begin{array}{cccc} k_{e}N_{e}\mathrm{wJ}-\frac{1}{\tau p} & k_{e}N_{e}J & -4k_{e}N_{i}w_{i1} & -4k_{e}N_{i}w_{i2}\\ k_{e}N_{e}J & k_{e}N_{e}\mathrm{wJ}-\frac{1}{\tau p} & -4k_{e}N_{i}w_{i2} & -4k_{e}N_{i}w_{i1}\\ 4{\mathrm{qk}}_{i}N_{e}J & 4k_{i}N_{e}J & -4k_{i}N_{i}\mathrm{gJ}-\frac{1}{\tau p} & -4k_{i}N_{i}\mathrm{gJ}\\ 4k_{i}N_{e}J & 4{\mathrm{qk}}_{i}N_{e}J & -4k_{i}N_{i}\mathrm{gJ} & -4k_{i}N_{i}\mathrm{gJ}-\frac{1}{\tau p} \end{array}\right)\\ B=\left(\begin{array}{ccccc} k_{e}\sqrt{c/\tau }\sigma_{i} & 0 & 0 & 0 & k_{e}\sqrt{\left(1-c\right)/\tau }\sigma_{i}\\ 0 & k_{e}\sqrt{c/\tau }\sigma_{i} & 0 & 0 & k_{e}\sqrt{\left(1-c\right)/\tau }\sigma_{i}\\ 0 & 0 & 0 & 0 & k_{i}\sqrt{1/\tau }\sigma_{i}\\ 0 & 0 & 0 & 0 & k_{i}\sqrt{1/\tau }\sigma_{i} \end{array}\right) \end{array}$$ (10)

In this formalization, the inhibitory weights from *I*_1_ and *I*_2_ to *E*_1_ and *E*_2_ are also dynamic and operate on a much slower timescale than the dynamics of the firing rates on the left side of Eq. 9. To obtain the governing equations for individual weight dynamics, we assume that these weights, which also play a role in shaping the dynamics of neuronal activities in Eq. 10, are piecewise constant (separation of timescales).

To obtain the slow dynamics of the inhibitory weights onto the excitatory neurons (*w*_*i*1_ and *w*_*i*2_), as suggested in (*25*), the cross-correlation function between the presynaptic (inhibitory) and postsynaptic (excitatory) firing rates, as well as the firing rates of the excitatory and inhibitory neurons at each moment in time are required

$$\begin{array}{c} {\overset{\cdot }{w}}_{i1}=\int_{-\infty }^{+\infty }{\mathrm{pv}}_{\text{stdp}}\left(\hat{t}\right)\left\{C\left[I_{1}\left(t\right),E_{1}\left(t+\hat{t}\right)\right]+\left[\left(r_{e}^{*}-r_{e}^{\text{target}}\right)r_{i}^{*}\right]\right\}\;d\hat{t}\\ {\overset{\cdot }{w}}_{i2}=\int_{-\infty }^{+\infty }{\mathrm{pv}}_{\text{stdp}}\left(\hat{t}\right)\left\{C\left[I_{1}\left(t\right),E_{2}\left(t+\hat{t}\right)\right]+\left[\left(r_{e}^{*}-r_{e}^{\text{target}}\right)r_{i}^{*}\right]\right\}\;d\hat{t} \end{array}$$ (11)

where the symmetric STDP function that defines *pv*_stdp_(*t*) is exp(−∣*t*∣/τ_stdp_). The functions $C\left[I_{1}\left(t\right),E_{1}\left(t+\hat{t}\right)\right]$ and $C\left[I_{1}\left(t\right),E_{2}\left(t+\hat{t}\right)\right]$ are the cross-covariance functions between *I*_1_ and *E*_1_, and *I*_1_ and *E*_2_, respectively, and are functions of $\hat{t}$ . The variables $r_{e}^{*}$ and $r_{i}^{*}$ are the solutions for the average firing rates of the neurons, which also depend on the weights *w*_*i*1_ and *w*_*i*2_, and are functions of time, but the homeostasis provided by the inhibitory plasticity ensures that the average firing rates of the E neurons in *E*_1_ and *E*_2_ converge to the target value $r_{e}^{\text{target}}$ . Because of this dependence on time, and also the dependence of the rates and synaptic weights on one another, the whole set of Eqs. 9 and 11 have to be solved self-consistently.

According to Eq. 11, to understand the dynamics of weight evolution for *w*_*i*1_ and *w*_*i*2_, we need to first evaluate the covariance functions between the E and I average neuronal dynamics, and then multiply them by the STDP function provided by p*v*_stdp_, which is composed of two exponential functions. It is known from (*65*) that for coupled Ornstein-Uhlenbeck (OU) processes, the expected covariance matrix can be obtained as follows

$$C\left(\hat{t}\right)=<X\left(t\right),X\left(t+\hat{t}\right)>=\left\{\begin{array}{cc} \text{exp}\left(A\hat{t}\right)Q & \text{if}\;\hat{t}>0\\ Q\text{exp}\left(-A^{T}\hat{t}\right) & \text{if}\;\hat{t}<0 \end{array}\right.$$ (12)

where *Q* is the solution to the following Lyapunov equation, which incorporates the correlation structure of the input in matrix B (defined in Eq. 10)

$$\mathrm{AQ}+{\mathrm{QA}}^{T}=-{\mathrm{BB}}^{T}$$

The challenge is to write the weight dynamics (Eq. 11) in a self-consistent way such that it only depends on the weights of the network and the correlation structure in the input written as a function of coupling weights. Since the right-hand side of Eq. 11 is integrals of exponential functions (coming from p*v*_stdp_) multiplied by a covariance function, we can evaluate the integral in the Laplace domain. For that, first, we need to find the Laplace transforms of the covariance functions in Eq. 12. Since the impulse response of the general system $\overset{\cdot }{y}=A\;y$ is exp(*A t*) in the time domain, and (*s***I** − *A*)^−1^ in the Laplace domain (*s* is the complex variable defined in the Laplace transform, and **I** is the identity matrix), the matrix exponentials in Eq. 12 can easily be replaced by (*s***I** − *A*)^−1^ and (*s***I** + *A^T^*)^−1^, respectively

$$C\left(s\right)=\left\{\begin{array}{cc} {\left(sI-A\right)}^{-1}Q & \text{if}\;\hat{t}>0\\ Q{\left(sI+A^{T}\right)}^{-1} & \text{if}\;\hat{t}<0 \end{array}\right.$$ (13)

where the element on row *i* and column *j* of matrix *C*(*s*) will be represented by *C*_*i*,*j*_(*s*). The covariance function between the E and I populations for all positive and negative lags can therefore be expressed as follows

$$\begin{array}{c} C\left(I_{1},E_{1}\right)\left(s\right)={\left[{\left(sI-A\right)}^{-1}Q\right]}_{3,1}+{\left[Q{\left(sI+A^{T}\right)}^{-1}\right]}_{3,1}\\ C\left(I_{1},E_{2}\right)\left(s\right)={\left[{\left(sI-A\right)}^{-1}Q\right]}_{3,2}+{\left[Q{\left(sI+A^{T}\right)}^{-1}\right]}_{3,2} \end{array}$$ (14)

This can be used to evaluate the integrals in Eq. 11 because the integral of any function from *t* = 0 to *t* = ∞ can be calculated by evaluating the function at *s* = 0 in the (one-sided) Laplace domain. It is also known that the multiplication of a linear system with an exponential function is equivalent to a shift operator in the Laplace domain. In other words, for the STDP function in the form of exp(−*t*/τ_stdp_), the integrals in Eq. 11 in the Laplace domain should be evaluated at *s* = 1/τ_stdp_. With this, Eqs. 11 and 12 can be rewritten as

$$\begin{array}{c} {\overset{\cdot }{w}}_{i1}={\left\{{\left[\left(s+\frac{1}{\tau }\right)I-A\right]}^{-1}Q\right\}}_{3,1}{|}_{s=0}+{\left\{Q{\left[\left(s+\frac{1}{\tau }\right)I+A^{T}\right]}^{-1}\right\}}_{3,1}{|}_{s=0}\\ +2\tau \left[r_{e}\left(t\right)-r_{e}^{\text{target}}\right]r_{i}\left(t\right)\\ {\overset{\cdot }{w}}_{i2}={\left\{{\left[\left(s+\frac{1}{\tau }\right)I-A\right]}^{-1}Q\right\}}_{3,2}{|}_{s=0}+{\left\{Q{\left[\left(s+\frac{1}{\tau }\right)I+A^{T}\right]}^{-1}\right\}}_{3,2}{|}_{s=0}\\ +2\tau \left[r_{e}\left(t\right)-r_{e}^{\text{target}}\right]r_{i}\left(t\right) \end{array}$$ (15)

where *r_e_*(*t*) and *r_i_*(*t*) are evaluated recursively and the whole system of equations above is solved in discrete domain. Starting from identical initial conditions for *w*_*i*1_(0) and *w*_*i*2_(0), and the corresponding solutions for *r_e_*(0) and *r_i_*(0), the weight dynamics of *w*_*i*1_(*t*) and *w*_*i*2_(*t*) can be obtained iteratively. Since the matrices *A* and *A^T^* include the dynamical variables *w*_*i*1_ and *w*_*i*2_, we will end up with a set of coupled ordinary differential equations that describe the evolution of these variables. This results in a state space plot for the dynamics of these weights (Fig. 2).

### Mean-field theory neglecting the neuronal correlations

In our mean-field theory for the synaptic weight dynamics (Eq. 11), we had two contributing terms: one term that considered the covariance between the E and PV neurons, and a second term that considered the product of the E and PV average population firing rates. To study the effect of the covariance term on the emergence of the structural tuning, we removed the contribution of the covariance functions from Eq. 11. In other words, we considered the following equations for the weight dynamics

$$\begin{array}{c} {\overset{\cdot }{w}}_{i1}=\int_{-\infty }^{+\infty }{\mathrm{pv}}_{\text{stdp}}\left(\hat{t}\right)\left\{\left(r_{e}^{*}-r_{e}^{\text{target}}\right)r_{i}^{*}\right\}d\hat{t}\\ {\overset{\cdot }{w}}_{i2}=\int_{-\infty }^{+\infty }{\mathrm{pv}}_{\text{stdp}}\left(\hat{t}\right)\left\{\left(r_{e}^{*}-r_{e}^{\text{target}}\right)r_{i}^{*}\right\}d\hat{t} \end{array}$$ (16)

The results of this variant of the mean-field model, ignoring the covariance terms, are shown in Fig. 3, B to D.
