# Supplementary material for: Emergence of co-tuning in inhibitory neurons as a network phenomenon mediated by randomness, correlations, and homeostatic plasticity
Source: Sci Adv. 2024 Mar 20;10(12):eadi4350. doi: 10.1126/sciadv.adi4350 (PMC12697572; doi:10.1126/sciadv.adi4350)
Supplement: Supplementary file 1 — Figs. S1 to S7 Table S1 [file sciadv.adi4350_sm.pdf]

Science Advances
AAAS

Supplementary Materials for

Emergence of co-tuning in inhibitory neurons as a network phenomenon mediated by randomness, correlations, and homeostatic plasticity

Fereshteh Lagzi and Adrienne L. Fairhall

Corresponding author: Fereshteh Lagzi, lagzi@uw.edu

Sci. Adv. **10**, eadi4350 (2024)
DOI: 10.1126/sciadv.adi4350

This PDF file includes:

Figs. S1 to S7
Table S1

Supplemental Figures

Network with Hebbian E and homeostatic PV-to-E plasticity

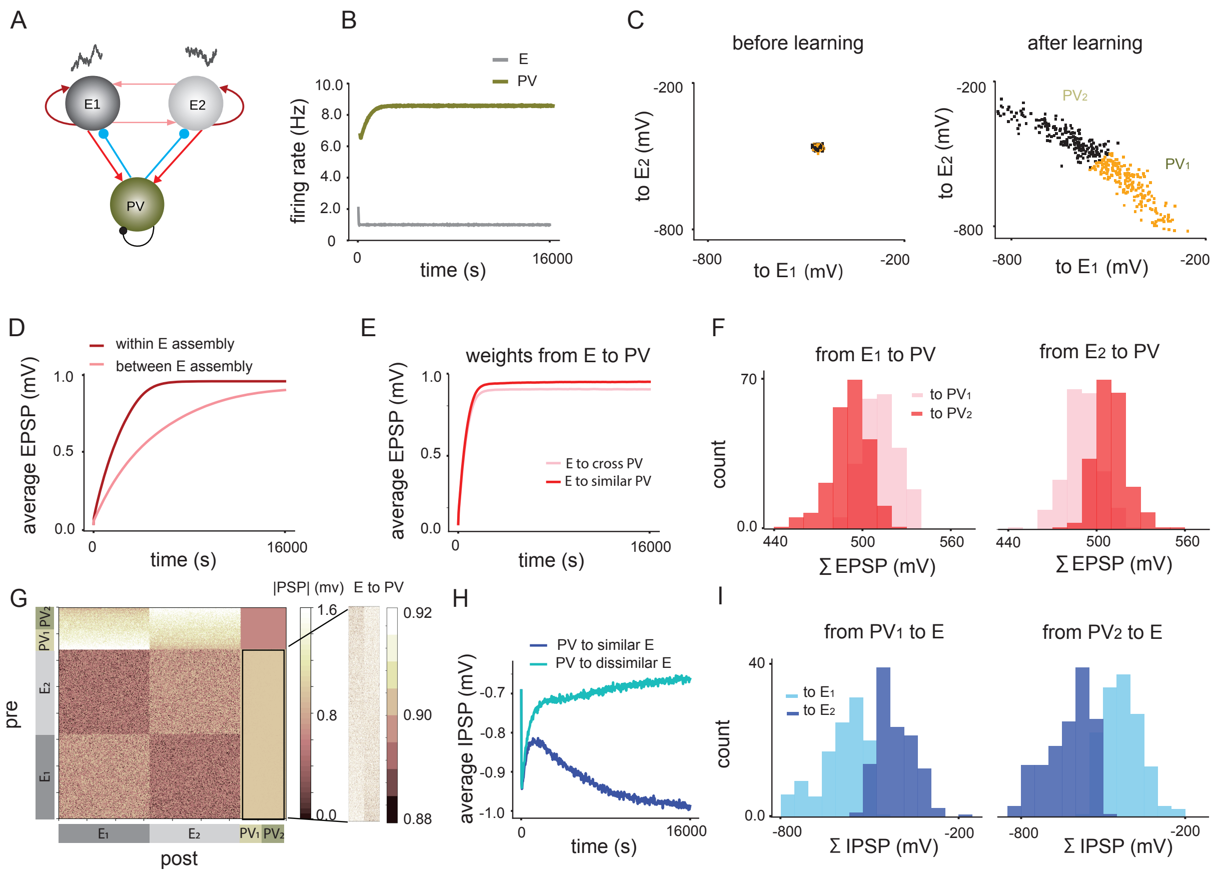

Fig. S1: Emergence of tuned PV to E populations in a fully plastic network with fixed PV to PV weights. **A**: Network initially composed of two E populations with a stronger probability of connections ($p = 0.15$) within each population, distinct shared input sources, and one PV population. All synapses followed alpha PSC dynamics with a synaptic time constant of 0.5 ms. Initial EPSP amplitudes were drawn from a lognormal distribution. **B**: Average neuronal firing rates of the E (homeostatic target rate was set to 1 Hz) and PV populations converge toward their steady-state values. **C**: Sum of IPSP projections of all PV neurons to E_1 and E_2 assemblies (outgoing PV measure in units of mV) define 2D vectors that are all very close to

each other (left). After learning, some PV neurons develop larger total IPSP sums to one of the E assemblies (right). PV neurons with larger IPSP sum to E_1 are labeled as PV_1, and the rest of the PV neurons are labeled as PV_2 neurons. **D**: Excitatory weights within and between E assemblies converge towards their maximum allowed value of 1 mV, following multiplicative Hebbian rule in (*30, 31*). **E**: Excitatory weights from E to PV neurons follow multiplicative Hebbian STDP, and heterogeneous probability of connections (50% of randomly chosen E neurons target PV neurons following a Bernoulli distribution with $\mathrm{p_{ei}} = 0.56$ and the rest of the E neurons connect to random PV neurons with $\mathrm{p_{ei}} = 0.8$). As a function of time, E_1 to PV_1 weights become slightly stronger than E_1 to PV_2 weights. This, consequently, plays a role in assigning PV clusters. **F**: left: Strong projections from E_1 to PV_1 and relative weaker projections from E_1 to PV_2 (also by symmetry, right: strong projections from E_2 to PV_2 and relative weaker projections from E_2 to PV_1) are the main drive of PV tuning. **G**: The entire connectivity matrix reflects the organization of the synaptic weights in the network with multiplicative Hebbian excitatory and homeostatic PV-to-E plasticity. A zoom into the E-to-PV weights is shown on the right side of G. Patterns of reciprocal connections between E and PV subnetworks are apparent. **H**: Average weight evolution for the connections from PV_1 to E_1 (more negative weights indicate stronger connections) and to E_2. **I**: Distribution of PV to E_1 and E_2 assemblies indicate that PV_1 neurons projected more strongly to E_1 neurons.

Principal Component Analysis for dimensionality reduction

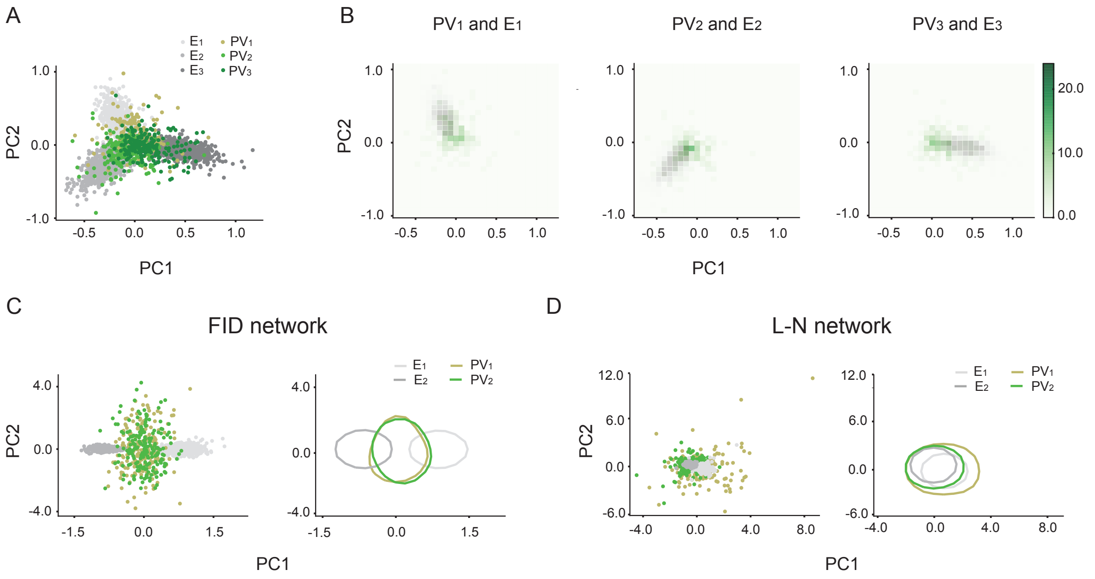

Fig. S2: PCA for dimensionality reduction. **A**: For the data presented in Fig. 4I, E and PV neuronal activities were projected in a low-dimensional PC subspace defined by the first 2 PC components. E neurons form distinct clusters with little overlap. PV neurons belonging to different subnetworks have a substantial overlap in the PC space, which indicates their broad tuning on a single-neuron level. Some PV neurons are sharply tuned. **B**: Distributions of co-tuned E and PV neuronal activities in separate plots show broad tuning of PV neurons on individual levels but some degree of tuning on a population level for the data presented in Fig. 4I. **C**: 2-dimensional PC projection of the data presented in Fig. 6D (FID network) reflects well-separated E subnetwork activity for E_1 and E_2, but substantial overlap between PV_1 and PV_2. The contours of the distribution of the data are shown on the right plot. **D**: Projection of the data for the L-N network shown in Fig. 6D onto the first 2 dimensions in the PC space illustrates a considerable overlap between the activity of the excitatory neurons in different clusters. PV neuron activity is slightly biased towards its co-tuned excitatory subnetwork. Contour plots of the distribution of the data points in the PC space are shown on the right panel.

Labeling PV neurons based on their maximum summed IPSP projections onto E assemblies (outgoing PV measure)

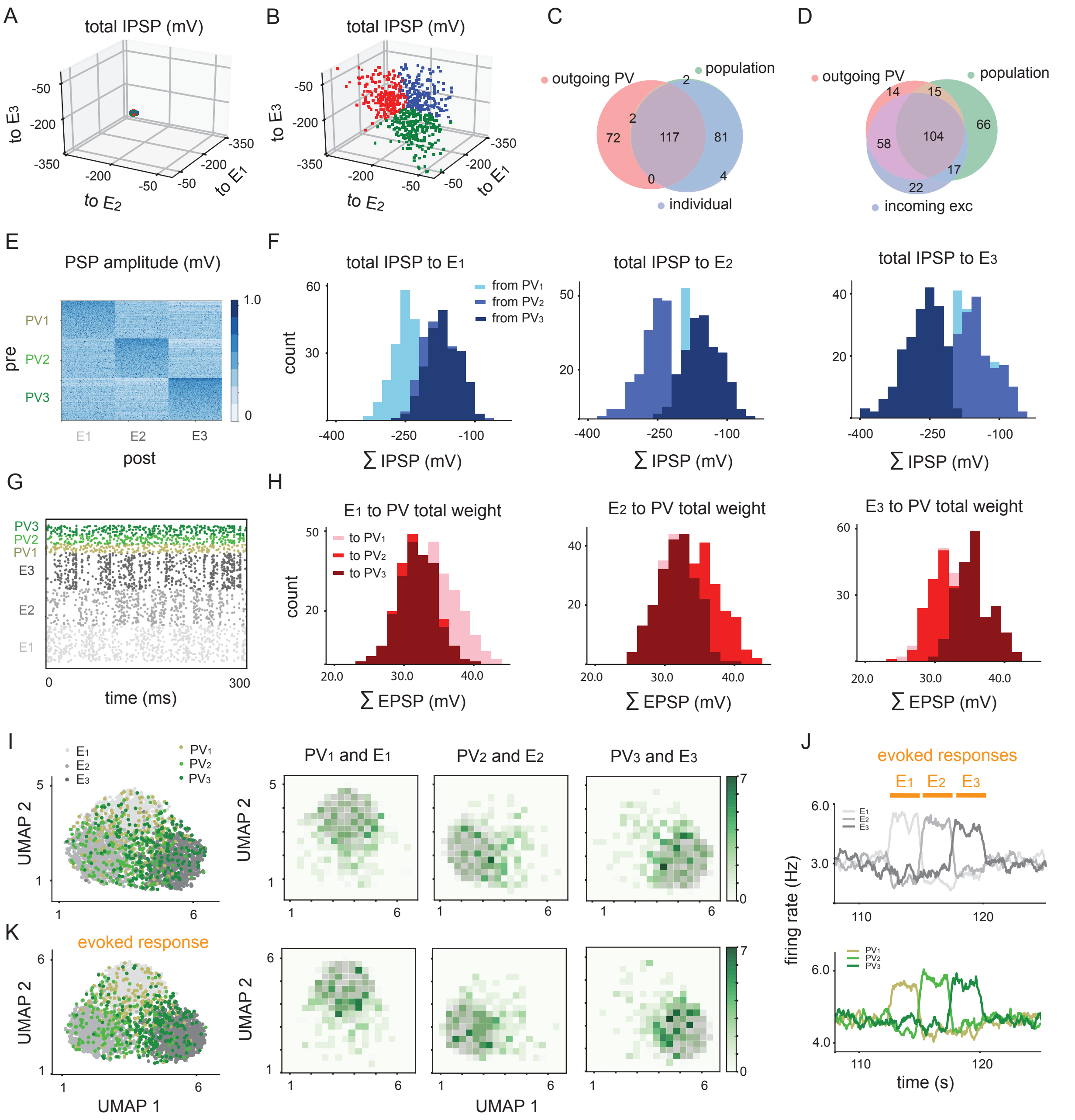

Fig. S3: Labeling PV neurons based on their maximum outgoing summed IPSP projections (outgoing PV measure) in the evoked state. **A**: The sum of the IPSP amplitudes for

the projection weights from single PV neurons to all neurons in E_1, E_2, and E_3 form one small 3-dimensional cluster of vectors, before plasticity operates. Colors are inherited from B after the assignment of feature preferences. **B**: After plasticity operation, PV neurons divide into 3 distinct groups with more preference to connect strongly to one of the excitatory assemblies: blue: preference to strongly connect to E_1; red: preference to strongly connect to E_2; green: preference to strongly connect to E_3. **C**: Venn diagram for the labeling of PV neurons that are assigned to PV_1 based on 3 measures: outgoing PV, population similarity, and individual similarity. The overlap between the last two measures is almost 100%. **D**: Venn diagram for the labeling of PV neurons that are assigned to PV_1 based on 3 measures: outgoing PV, population similarity, and incoming E. There is a large overlap between any two measures. **E**: Connectivity matrix from PV to E neurons after labeling PV neurons based on the outgoing PV measure. **F**: Distribution of the total IPSP weights from neurons in individual PV populations to E_1 indicate that PV_1 has the highest preference of connection to E_1; hence these group of PV neurons were labeled as PV_1 (left). Similarly, PV_2 (PV_3) neurons have the strongest projections onto E_2 (E_3). **G**: Raster plot of neuronal activities for the last 300 ms of ongoing plasticity. **H**: Total EPSP projections from individual neurons in E_1 onto the assigned PV populations indicate that E_1 had a stronger total projection weight onto PV_1 neurons (left). A similar relation holds for other excitatory assembly projections: e.g summed EPSP weights from E_3 onto PV_3 were more skewed towards bigger values (right). **I, K**: 2-dimensional UMAP projection of the neuronal activities for 7.5 seconds of simulation time according to their distance in the high dimensional space for the spontaneous (I) and evoked (K) state. Planar density of co-tuned excitatory and PV neurons are plotted separately for each feature defined by shared correlated input. **J**: Evoked responses of the E and PV subnetworks as a result of sensory stimulation of individual E assemblies.

Effect of wide vs narrow E to PV weights in shaping the relationship between EPSP and IPSP

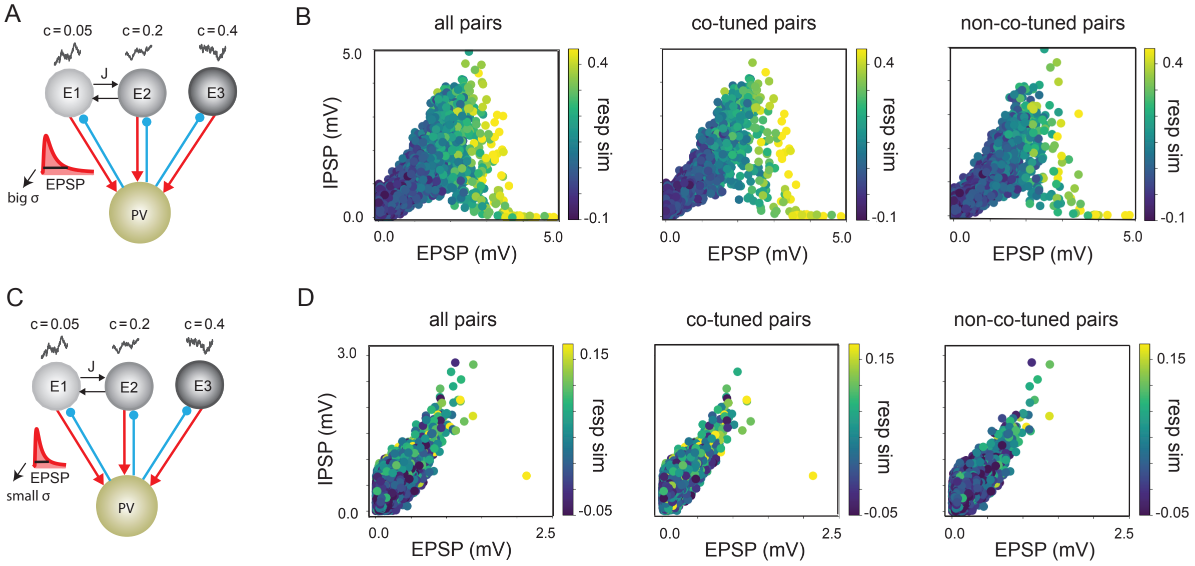

Fig. S4: Effect of high and low variance in E to PV connection strength on the reciprocal PV to E connection weights. **A**: Network schematic for connections with high variance (big σ) for the log-normal distribution. **B**: Scatter plot of all EPSP and IPSP, for pairs with reciprocal connections, with pairwise response similarity as color codes for the network in A. Co-tuned and non-co-tuned pairs are plotted separately. Very high values of EPSP result in decreased IPSP amplitudes for large values of EPSP. **C**: Network schematic for connections with low variance (small σ) for the log-normal distribution. **D**: EPSP and IPSP relationship and response similarity between pairs of reciprocally connected PV and E neurons for low variance connections follows a linear trend.

Pairwise versus population measures can result in different conclusions for the tuning of PV neurons

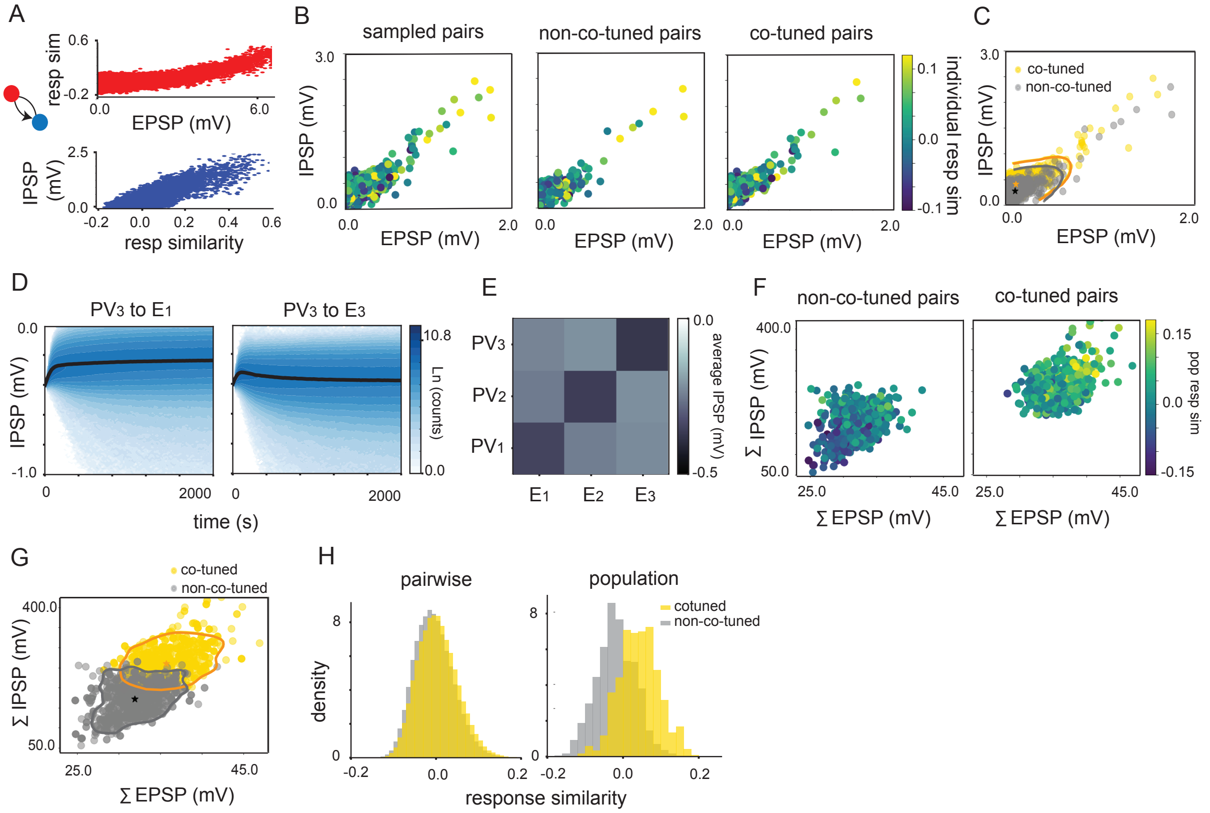

Fig. S5: Pairwise and population measures for defining PV subnetworks based on the outgoing PV measure. **A**: For reciprocally connected E and PV neurons in the spontaneous state, response similarity increases with increasing values of EPSP. The strength of IPSP increases monotonically with the pairwise correlation coefficient. **B**: left: EPSP and IPSP values for 2000 sample of reciprocally connected pairs. middle, right: Reciprocal EPSP and IPSP values for pairs belonging to non-co-tuned populations and similarly tuned populations. **C**: Distribution of co-tuned and non-co-tuned pairs. Contour lines define the area that contains 95% of the data for each group. Mean EPSP and mean IPSP for each group are indicated by a star (orange: co-tuned group, gray: non-co-tuned group). **D**: Evolution of IPSP distributions from PV_3 neurons

to E_1 (non-co-tuned) and E_3 (co-tuned) neurons as a function of time. **E**: Average connectivity matrix for the weights from PV subnetworks onto excitatory assemblies. **F**: Summed IPSP of individual PV neuron projection onto co-tuned (right) and non-co-tuned (left) E neurons as a function of the sum of the EPSP received by the E assemblies. The color bar defines the cosine similarity of the PV neurons with the average excitatory populations. **G**: 2D scatter plots for co-tuned and non-co-tuned pairs and contour lines defining regions in the space with 95% of the data for each case. Stars represent the mean values of the total EPSP and IPSP for each case. Summed IPSP as a function of summed EPSP, on average, takes bigger values when the sum of the EPSPs are large (neurons are similarly tuned). **H**: Density distribution of cosine similarity between pairs of E and PV neurons belonging to co-tuned or non-co-tuned E assemblies are not distinguishable; however, if response similarity between individual PV neurons and population responses of different E assemblies are considered, the distributions of the response similarities for the co-tuned and non-co-tuned PV neurons become more separable.

Effect of heterogeneity on PV tuning and feature selectivity

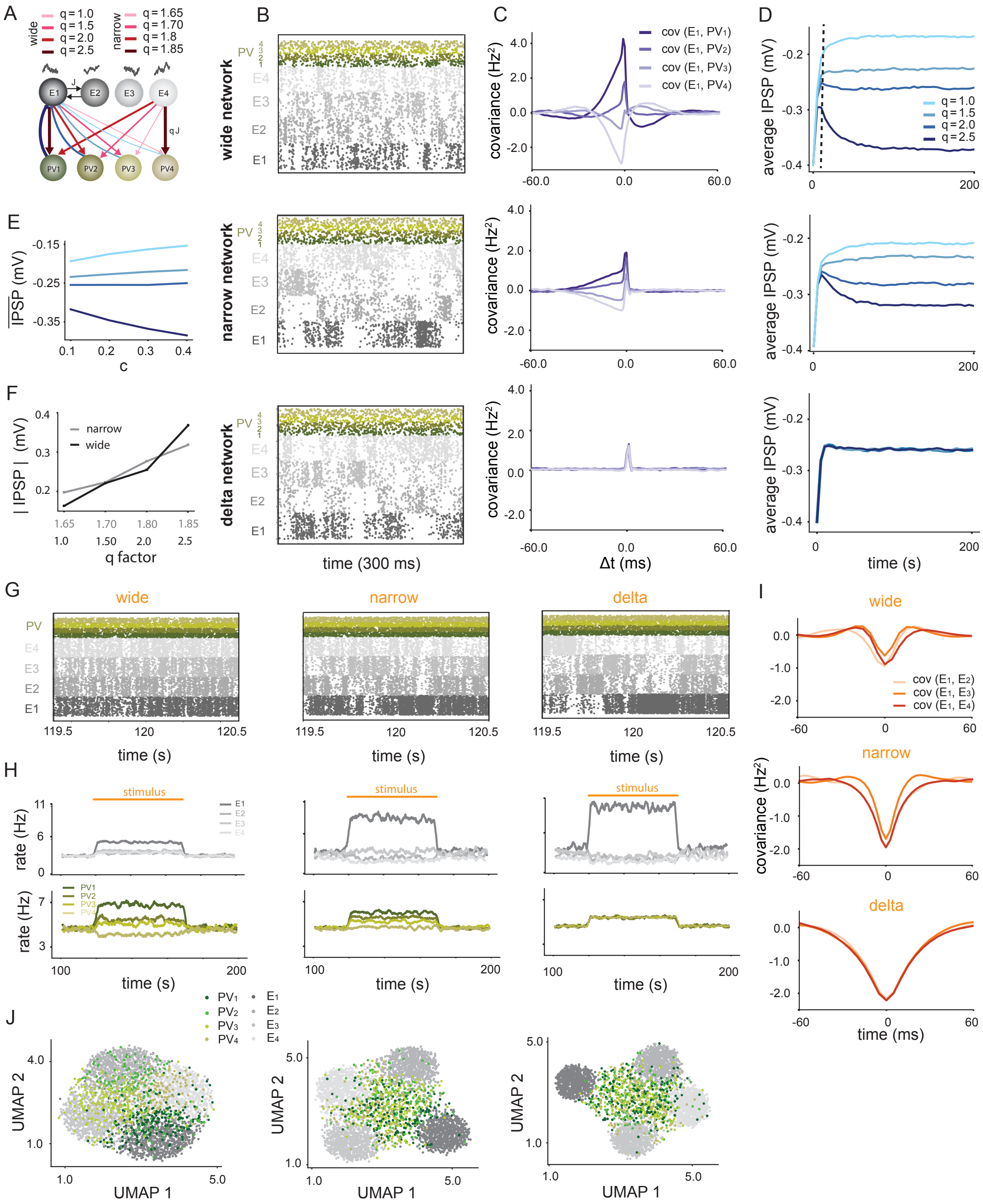

Fig. S6: Effect of heterogeneity in EPSP values for E-PV synapses and its impact on feature selectivity of E neurons. **A**: Network with 4 similar excitatory assemblies and a profile of connectivity to PV subnetworks with varying EPSP amplitudes modified by (q = 1.0, 1.5, 2.0, 2.5) for the wide network, by (q = 1.65, 1.70, 1.80, 1.85) for the narrow network, and all q = 1.75 for the delta network. All reciprocal IPSP amplitudes were identical at the beginning of the simulation. **B**: Raster plot for the wide network (top), narrow network (middle) and delta network (bottom) after 1000 seconds of training indicates high correlations between excitatory neurons within each assembly for the narrow and delta network. **C**: Covariance function between average PV_i and average E_1 neuronal firing rates for the wide network (top), narrow network (middle) and delta network (bottom). **D**: Evolution of IPSP amplitudes from PV_1 through PV_4 onto E_1 assembly. Smaller values of q in the corresponding EPSP amplitude from E_1 result in weaker reciprocal projection from the PV subnetwork back onto E_1. **E**: Steady state amplitude of the IPSP corresponding to different reciprocal q values for the EPSP of the projecting connection as a function of increasing the value of input correlation c. The distance between the IPSP amplitudes increases with increasing c. **F**: Monotonic relationship between IPSP and EPSP amplitudes as a function of increasing q for the wide (black) and narrow (gray) network. **G**: Raster plots of the networks before and after the onset of sensory input to E_1 at $\mathrm{t} = 120\,\mathrm{s}$. **H**: Average neuronal firing rates for the excitatory (top) and PV (bottom) populations in 100 seconds interval for which sensory input evokes E_1 activity from $\mathrm{t} = 120$ s to $\mathrm{t} = 170$ s. **I**: Cross-covariance between E_1 and other excitatory assemblies take more negative values (increased competition) as the distribution of EPSPs from E to PV gets narrower. **J**: from left to right: 2-dimensional UMAP plots for the neuronal activities for the wide, narrow and delta networks when E_1 receives specific sensory input. Reduced dimension responses show more separable activities for the E neurons as the level of heterogeneity decreases, but less tuned PV to E neuronal activities.

Covariance between population firing rates for the fixed in-degree and log-normal network

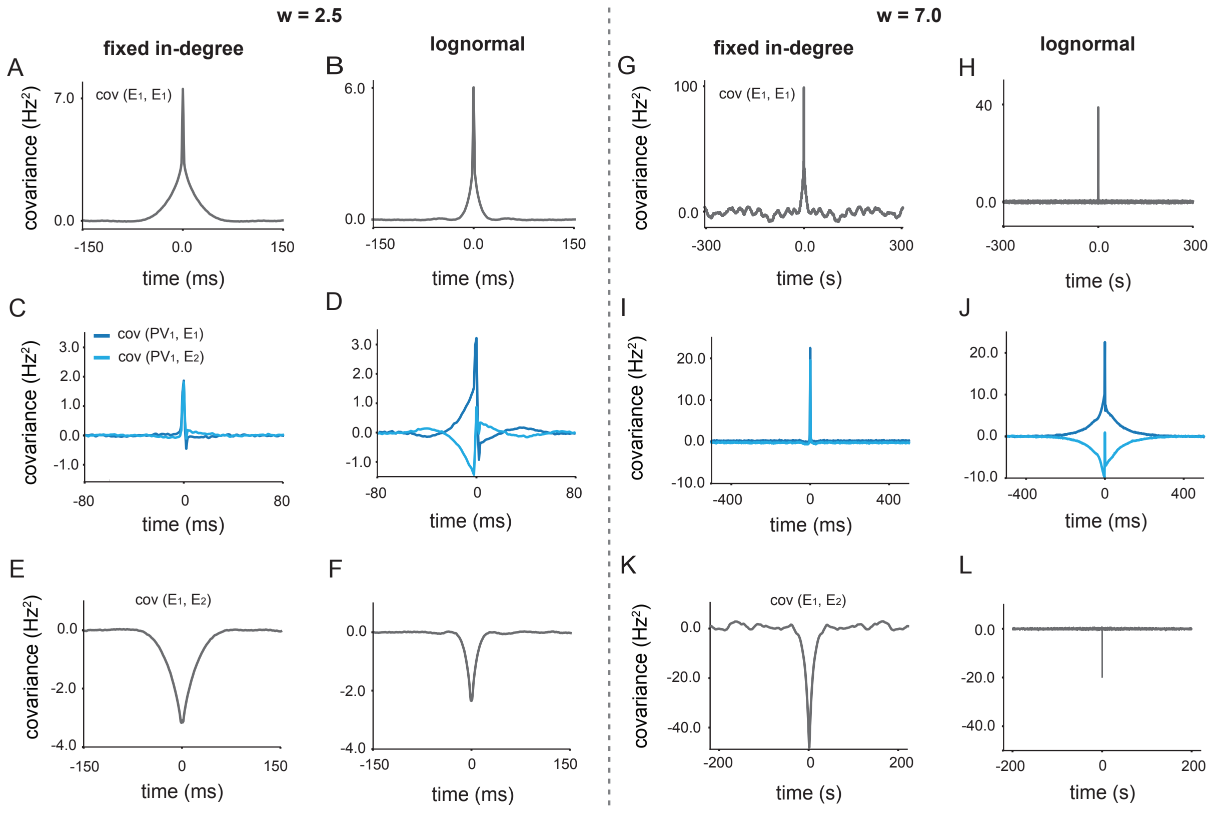

Fig. S7: Covariance between population firing rates in Fig. 7. **A, B**: Auto-covariance function for the FID and L-N network for $w = 2.5$. **C**: For the FID nework and $w = 2.5$, both covariance functions between PV_1 and the firing rate of E_1 and E_2 are almost identical. **D**: For the L-N network with lognormal E to PV distributions,PV_1 and E_1 are positively correlated, while PV_1 and E_2 are negatively correlated. **E, F**: Cross-covariance function between E_1 and E_2 indicates stronger competition between assemblies for the fixed in-degree network. **G, H**: Auto-covariance function of the E assemblies indicates very slow dynamics for the FID network when w was increased to 7.0. **I, J**: For $w = 7.0$, for the network with log-normal distribution, the covariance function between PV_1 and E_1 is strictly positive, while the covariance between

PV_1 and E_2 is strictly negative. These functions are identical for the FID network. **K, L**: Cross-covariance between the E populations reflects stronger competition between the E assemblies for the FID network, for $\mathrm{w} = 7.0$.

Table S1: Parameters used in network simulations for figures 1 to 7

Variable	Explanation	figure 1 and 7	figure 2 and 3	figure 4 and 5	figure 6
N_e	Number of E cells in each assembly	800	3200	800	800
N_i	Number of PV neurons	overall 400	overall 1600	200 per group	200 per group
J	EPSP amplitude for fixed connections (mV)	0.04	0.02	0.04	0.04
w	scalar for within assembly weights	2.5	2.5	2.5	2.5
g	Scalar for the fixed IPSP amplitudes	10.0	10.0	10.0	10.0
θ	Threshold for the membrane potential (mV)	20.0	20.0	20.0	20.0
v_{reset}	membrane potential reset level (mV)	10.0	10.0	10.0	10.0
v_{rest}	resting potential (mV)	0.0	0.0	0.0	0.0
τ	membrane potential time constant (ms)	20.0	20.0	20.0	20.0
C	Capacitance of the membrane potential (μ F)	250.0	250.0	250.0	250.0
p	probability of connection between E cells	10%	10%	10%	10%
p_{ie}	probability of connections from E to PV	40%	40%	90%	40%
p_{ei}	probability of connections from PV to E	40%	40%	40%	40%
p_{ii}	probability of connections between PV cells	40%	40%	40%	40%
τ_{stdp}	time constant of PV to E stdp (ms)	20.0	20.0	20.0	20.0
η_e	learning rate for E to E connections	supp: 0.000015	NA	NA	NA
η_{ep}	learning rate for E to PV connections	supp: 0.000005	NA	NA	NA
η_p	learning rate for PV to E connections	supp: 0.00005	0.00005	0.00005	0.00005
r^*	target homeostatic firing rate (Hz)	3.0	variable	3.0	3.0
c	percentage of shared input from the total input	30%	30%	variable	30%
σ	std of the external Poisson process (Hz)	$\sqrt{37500}$	$\sqrt{60000}$	E cells: $\sqrt{37500}$<br>PV cells: $\sqrt{24375}$	$\sqrt{30000}$
